# Revisiting Excitotoxicity in Traumatic Brain Injury: From Bench to Bedside

**DOI:** 10.3390/pharmaceutics14010152

**Published:** 2022-01-08

**Authors:** Daniela Baracaldo-Santamaría, Daniel Felipe Ariza-Salamanca, María Gabriela Corrales-Hernández, Maria José Pachón-Londoño, Isabella Hernandez-Duarte, Carlos-Alberto Calderon-Ospina

**Affiliations:** 1 Pharmacology Unit, Department of Biomedical Sciences, School of Medicine and Health Sciences, Universidad del Rosario, Bogotá 111221, Colombia; daniela.baracaldo@urosario.edu.co (D.B.-S.); maria.corrales@urosario.edu.co (M.G.C.-H.); mariajo.pachon@urosario.edu.co (M.J.P.-L.); isabella.hernandez@urosario.edu.co (I.H.-D.); 2 Medical and Health Sciences Education Research Group, School of Medicine and Health Sciences, Universidad del Rosario, Bogotá 111221, Colombia; daniel.ariza@urosario.edu.co; 3 GENIUROS Research Group, Center for Research in Genetics and Genomics (CIGGUR), School of Medicine and Health Sciences, Universidad del Rosario, Bogotá 111221, Colombia

**Keywords:** excitotoxicity, traumatic brain injury, novel therapeutics, NMDA receptor, GABA, vitamin B12, astrocyte, calcium, oxidative stress, neuroinflammation

## Abstract

Traumatic brain injury (TBI) is one of the leading causes of morbidity and mortality. Consequences vary from mild cognitive impairment to death and, no matter the severity of subsequent sequelae, it represents a high burden for affected patients and for the health care system. Brain trauma can cause neuronal death through mechanical forces that disrupt cell architecture, and other secondary consequences through mechanisms such as inflammation, oxidative stress, programmed cell death, and, most importantly, excitotoxicity. This review aims to provide a comprehensive understanding of the many classical and novel pathways implicated in tissue damage following TBI. We summarize the preclinical evidence of potential therapeutic interventions and describe the available clinical evaluation of novel drug targets such as vitamin B12 and ifenprodil, among others.

## 1. Introduction

Traumatic brain injury (TBI) is defined as the acquired cerebral disease that results from an external force in the form of mechanical, chemical, thermal, or electrical energy, or a combination of these [1]. These forces have the potential to cause structural harm to the cranial cavity, brain tissue, and blood vessels. Globally, TBI is one of the highest causes of morbidity and mortality. According to statistics from the United States, up to 43% of severe TBI survivors will probably suffer long-term disability, disrupting individual development [2]. The burden of TBI extends to the patient’s family and the health care system [3,4].

The incidence of TBI is notably high, affecting up to 69 million people worldwide per year, with an estimated annual incidence of 811 to 1507 cases per 100,000 people, depending on the region [5,6,7]. In 2018, the highest incidence was in North American Countries, the highest burden was in the Southeast Asia and Western Pacific regions, and low- and middle-income countries needed to improve their data availability [5]. Nonetheless, a recent, encouraging analysis showed that TBI incidence gradually decreased 4.4% between 1990 and 2017 in the US [8].

For therapeutic and pathophysiological purposes, the pathogenesis of TBI has been classically divided into two injury mechanisms. The initial external mechanical force (rotational or shear forces) is considered as the primary injury mechanism [9], which can be further classified as penetrating or non-penetrating, and can lead to epidural, subdural, and intracerebral hematoma, intraventricular hemorrhage, or diffuse axonal injury. Skull fractures and vasogenic edema are also consequences of primary injury and they cannot always be fully repaired, though they can be surgically mitigated [1,10]. The main secondary injury hallmarks of TBI are changes in neurotransmitter release and metabolism, intracellular calcium dysregulation [11], biochemical reagent overproduction [12], blood brain barrier (BBB) disruption [13], cellular lysis, cellular and cytotoxic edema [14,15], and inflammation [16,17,18].

Head elevation, hyperventilation, seizure prophylaxis, hyperosmolar therapy, induced coma, cooling, and decompressive craniectomy are the current pillars for the acute treatment of patients following TBI [19]. Regarding disability prevention in acute and chronic treatment after TBI, Sveen and colleagues showed that an immediate discharge to specialized rehabilitation therapy is related to better outcomes and independence level of the patient when compared to delayed discharge or no rehabilitation at all 12 months after a severe TBI [20]. Identified barriers regarding access to rehabilitation include limited resources, patient’s safety concerns, lack of support from family members, or health care provider restrictions [21].

Disability rates vary depending on the severity of the trauma, as well as the accuracy and quality of the treatment given and subsequent follow-up. Events related to the highest disability rates are firearm injury and fall [22]. The prevalence of disability has been estimated at 3.32 million to 5.3 million in the US [6,23]. Direct costs related to TBI have been estimated at 9.2 billion per year, and indirect costs related to missed work and loss of productivity are up to 51.2 billion dollars [6]. It is noteworthy that the greatest burden of disease comes from disability and loss of productivity. In that sense, it is mandatory to recognize therapeutic approaches to mitigate disability, or maybe even avoid it.

Excitotoxicity, understood as neural death secondary to the excessive release of excitatory neurotransmitters, is broadly described as a common pathway leading to cell death in many neuropathological conditions [24,25,26,27,28]. When it comes to TBI, excitotoxicity is promoted by an increase of glutamate concentrations released into the extracellular space due to cellular lysis, followed by a rise of positive charges within the cell [29]. Calcium (Ca^2+^) activates second messenger pathways, exacerbates oxidative stress (OS), and initiates programmed cell death [30]. These factors can act as enhancers and perpetuators of excitotoxicity.

In this paper, we will focus on revisiting the classical and novel pathophysiological pathways involved in TBI to adequately understand new potential drug therapies. We will describe, in detail, mechanisms such as NMDAr (*N*-methyl-d-aspartate receptor)-dependent excitotoxicity, mechanoporation, GABA (Gamma-Aminobutyric acid)-mediated excitotoxicity, and the role of protagonist processes in TBI such as neuroinflammation, BBB disruption, and OS. New therapeutic agents regarding these pathways will also be presented and critically assessed to propose which of them could have a positive impact to avoid or mitigate secondary lesions after TBI and therefore alleviate the subsequent long-term disability and disease burden.

## 2. Mechanisms for Excitotoxicity

NMDAr activation is a major contributor to the enhancement of excitotoxicity following TBI. To better understand its activation, a review of the receptor structure is needed. NMDA receptors are formed by the assembly of seven subunits divided into three main subfamilies (GluN1, GluN2A-D and GluN3A-B) [31], each encoded by seven different genes, to form a final heterotetrametric structure that forms an ion channel that is permeable to Na^+^, K^+^ and Ca^2+^. It has two obligatory subunits: the GluN1 subunit which binds glycine and d-serine and a GluN2 subunit which binds glutamate; and/or two GluN3 (A or B) subunits [32]. The GluN2 subunit has a great influence on the EC_50_ (half maximal effective concentration) of glutamate depending on which of the four subunits is expressed (A-D). NMDA receptors that contain one GluN1 and two GluN2D subunits have an EC_50_ five-fold lower for glutamate than those receptors with two GluN2A, 2B, or 2C subunits [31]. Similarly, some subunits have a higher permeability to Ca^2+^ and express a higher sensitivity to the magnesium block (GluN1/2A, and GluN1/2B) [31]. Interestingly, NMDA receptors containing the GluN2B subunit have been implicated in aiding the spread of excitotoxicity among neighbor cells in cultured hippocampal neurons. In contrast, the same study suggested that NMDA receptors containing GluN2A favor neuroprotective signaling mechanisms [33].

In addition, NMDA receptors are capable of binding small ligands, especially to the amino terminus of the receptor subunits. These ligands can act as allosteric modulators, which differ from NMDA antagonists in that they can modulate the receptor activity without altering its biological functions [34], and are thus a potential drug target. The GluN2B subunit has three different binding sites for both negative and positive allosteric modulators. They interact with zinc and ifenprodil, producing an allosteric inhibition of the receptor [34].

### 2.1. Glutamate-Mediated Activation

Glutamate has been addressed as the most important neurotransmitter and it is involved in many processes including synaptic connectivity, cellular ionic homeostasis, long term potentiation, and even hormonal release [35,36]. All these cellular processes take place mainly through the regulation of intracellular concentrations of Ca^2+^ ([Ca^2+^]_i_). When studied in traumatic brain injury, the extracellular concentrations of glutamate rise [37] as a consequence of cellular lysis. The later aberrant efflux of glutamate from neurons and glia ends up worsening the scenario. High concentrations of glutamate are harmful, as it can over-activate synaptic NMDAr as well as extrasynaptic glutamate receptors [38].

Different studies have pinpointed NMDAr activation as the main cause of excitotoxicity after TBI [37,39]. NMDAr activation precedes Ca^2+^ and Na^+^ entry into intracellular spaces. High concentrations of [Ca^2+^]_i_ activate second messenger pathways, caspases and calpain proteins, and the release of neurotransmitter vesicles, catabolic enzymes as phospholipases, proteases and endoproteases [40]. An indirect way to measure glutamate concentration is by recording cortical spreading depolarizations, which reach their maximum 48 h after trauma [41]. Essentially, after a TBI there is an increase in extracellular glutamate concentrations and an upregulation in ionotropic glutamate receptors such as AMPA (α-amino-3-hydroxy-5-methyl-4-isoxazolepropionic acid) and NMDA [40]. Together, these factors promote Ca^2+^ and Na^+^ entry into the neuron. Once Ca^2+^ and Na^+^ are free in the neuron’s cytosol, different processes will occur. Two main organelles, the mitochondria and the endoplasmic reticulum, play an important role in the regulation of [Ca^2+^]_i_.

Furthermore, a study by Lee and colleagues [42] demonstrated the expression of NMDA receptors on the astrocytic membrane, accompanied by an increase in glutamate release following high concentrations of intracellular calcium. With this being said, it can be stated that, as with neurons, higher concentrations of extracellular glutamate activate NMDAr, thereby augmenting calcium influx into the astrocyte. Higher calcium concentrations will override the astrocyte’s mechanism of glutamate reuptake and transformation, thus potentiating the chain reaction leading to excitotoxicity.

### 2.2. Mechanical Activation of NMDAr and Mechanoporation of Neuronal Membranes

Mechanical NMDA activation can occur secondary to lipid bilayer tension, allowing Ca^2+^ to enter the neuron in the absence of NMDA agonists [43]. The physiological response of rat astrocytes to shear stress confirmed the mechanosensitive properties of NMDA in the absence of agonists that effectively remove the Mg^+^ blockade [44], and therefore linking the shearing and stretching forces produced in TBI to glutamate-independent excitotoxicity. Some studies have attributed this mechanosensitive nature of the receptor to the GluN2B subunit, specifically the serine 1323 residue in the C-terminus [45].

Mechanoporation refers to the formation of pores in the cell membrane induced by mechanical forces [46,47,48]. These pores form in milliseconds because of membrane stretching, which leads to the inhibition of action potential generation [49]. Mechanoporation alters the permeability of the membrane and allows the flow of ions and molecules that lead to cell death. This mechanism of injury has been demonstrated through indirect measurements using different markers that show alteration of membrane permeability [50]. Understanding the creation of pores in the membrane of neurons following brain trauma led to the conclusion that Ca^2+^ could enter the cell by the same pathway as the fluorescent markers evaluated in some studies [50], and could therefore exert direct excitotoxicity without the need for a transporter. In addition, the extent of membrane damage depends on the mechanism of injury: in three-dimensional neural cultures analyzing cell survival and membrane permeability, it was shown that cells were more sensitive to bulk shear deformation than compression [51].

It has been proposed that the creation of these membrane pores can occur in two stages [51]: the initial injury that causes stretch and shearing forces, therefore creating mechanical damage to the membrane; and a secondary injury that consists of membrane degradation through the activation of degradative enzymes (e.g., calpain) [51]. Interestingly, Ca^2+^ influx secondary to mechanoporation has been postulated in some studies as necessary for calpain activation in axons of cultured neurons [52]. Furthermore, following calpain activation, axonal microtubule damage ensues, causing impaired axonal transport [53].

Ultimately, excitotoxicity will lead to high [Ca^2+^]_i_, which is responsible for the tissue damage and cellular death that will be discussed in the next section.

### 2.3. Consequences of High Intracellular Calcium

In the mitochondria, elevated calcium concentrations will have different catastrophic repercussions, among which are the generation of free radicals and reactive oxygen species (ROS), protease and phospholipase activation, nitric oxide synthase (NOS) activation, and the generation of mitochondrial pores through which contents such as Cytochrome c will leak and promote the activation of caspases and nucleases, eventually leading to signaling and cellular death. ROS-induced OS is a major cause of neuronal death in the context of secondary injury after brain trauma, as it damages nucleic acids, proteins and lipids [54]. OS contributes to mitochondrial dysfunction and therefore energy depletion. In a study about OS in rats following mild and severe TBI, OS did not show a pattern in terms of proportionality between the energy of the trauma and the extent of the secondary injury. Additionally, OS was consistent across the areas of the brain without preference [55].

Another mechanism by which high [Ca^2+^]_i_ is detrimental to the cell is the acidification resulting from Ca^2+^ influx, as this acidification will activate the Na^+^-H^+^ exchanger, a pH-regulating mechanism that will decrease the activity of the Na^+^-Ca^2+^ exchanger, affecting the cell’s ability to pump calcium out [56].

Mitochondrial Ca^2+^ overload added to a densely ROS-concentrated environment will lead to the formation and opening of mitochondrial transition pores. Given the close proximity of the outer mitochondrial membrane with the outer endoplasmic reticulum, these pores will function as a Ca^2+^ efflux mechanism. Mitochondrial transition pores are not selective, meaning that although calcium is flowing out of the cell, other intra-mitochondrial components such as Cytochrome c will also leak out, activating a chain reaction and ultimately resulting in cell death [57]. Moreover, the continuous opening of mitochondrial transition pores will alter the mitochondrial membrane potential, leading to a decrease in ATP (adenosine triphosphate) production, thus resulting in mitochondrial destruction [58]. Additionally, as Ca^2+^ and Cytochrome c are being liberated, water and cytosolic solutes are entering the mitochondria, leading to swelling and, consequently, matrix rupture [59].

Caspases are a family of intracellular cysteine proteases involved in inflammatory processes and programmed cell death, and are activated by high [Ca^2+^]_i_ [60]. Caspase 1 cleaves acid residues, aspartic acid, and matures interleukin 1, which exacerbates damage [61,62]. As for Caspase 3, its activation will lead to the proteolysis of DNA-repairing proteins and the degradation of cytoskeletal proteins, eventually resulting in morphological changes that lead to apoptosis [63,64].

Calpains are Ca^2+^-activated neutral proteases involved in multiple pathways such as remodeling, cell signaling, synaptic plasticity, neuroprotection, learning, and programmed cell death [65,66]. Evidence suggests calpains are early regulators of cell death after TBI. Calpain activation is evident five hours after trauma in the dendrites, with later expression in the soma after 10 h [66]. Recent evidence shows calpain 1 and calpain 2 are directly implicated in cell death. Wang and colleagues [67] showed calpain-2 is responsible for NMDA-induced excitotoxicity through the activation of striatal-enriched protein tyrosine phosphatase (STEP) when extra synaptic NMDA receptors are activated in rat-cultured neurons. STEP activation mediated by calpain-2 results in p38 expression and, therefore, downstream cell death [65,68]. After TBI, high [Ca^2+^]_i_, OS, and ischemia, induce endoplasmic reticulum (ER) stress, which oversees membrane protein synthesis, folding, and quality control. When the ER is stressed, the unfolded protein response (UPR) is started by PERK (PKR-like ER kinase), ATF6 (activating transcription factor 6), and IRE-1 (inositol requiring enzyme 1) when they are dissociated from glucose-regulated protein/binding immunoglobulin protein (GRP78/BIP) [69]. PERK phosphorylates eukaryotic initiation factor 2α (eIF2α), which reduces protein synthesis, while IRE-1 promotes the UPR response, and cleaved ATF6 activates chaperones and intrinsic systems in charge of ER-associated degradation systems [70,71]. These pathways ultimately lead to C/EBP homologous protein (CHOP) upregulation, resulting in cellular apoptosis.

It is remarkable that high intracellular calcium is the common consequence of multiple damages sustained during TBI, such as the formation of cellular and mitochondrial membrane pores and ER stress, resulting in cellular death. Therefore, most therapies have focused on preventing or controlling the rise of [Ca^2+^]_i_.

### 2.4. Hippocampal Vulnerability to TBI

Following TBI, hippocampus-dependent cognitive impairment, such as memory loss, represents a significant burden for the patient and their family. The hippocampus is an interesting area because it is one of the specific regions of the brain where adult neurogenesis occurs [72] and where most animal studies in different TBI models (controlled cortical impact [73], fluid percussion [74] and stretch injury [75]) have shown the most vulnerability [76]. In particular, the subventricular zone of the lateral ventricle and the subgranular zone of the dentate gyrus (SGZ-DG) in the hippocampus have been linked to neurogenesis. Normally, these newborn neurons are continuously being generated. Approximately 700 new neurons are added every day to the hippocampus and then, as maturation occurs, they migrate and incorporate into the hippocampal circuit. The adult-born SGZ-DG cells differ from their mature counterparts in that they have a higher membrane resistance, lack glutamatergic input, and are depolarized by GABAergic input [77]. They have been linked to the consolidation of new memories, long-term spatial memory, cognitive flexibility, pattern separation and increased performance in behavioral tasks [78]. Similarly, they might be involved in the cognitive impairment seen in the chronic phase following TBI as there are studies that have shown the selective death of hippocampal newborn neurons after moderate brain trauma.

The distribution of degenerating neurons in the hippocampus of the mouse brain following moderate TBI was assessed by Gao and colleagues, who found that most of the degenerating hippocampal neurons were located in SGZ-DG [76]. Furthermore, another study [79] demonstrated that most of the neuron death that occurs in the hippocampus occurs within 24 h post-injury and continues for another 14 days. They also found that most of the dying cells in the SGZ-DG were immature. In addition, another study utilized an ionic fluorescein (FJP-fluoro-jade B) derivative reported to be a specific marker for degenerating neurons in young male rats subjected to controlled cortical impact [80]. By using the fluorescein marker, they found that there were FJP-positive neurons in the hippocampus, with higher expression in the moderate TBI group compared to the mild TBI group. The regional distribution of FJB staining was different according to the days post-injury and the severity of the TBI, and there was a direct relationship between the number of FJB-positive neurons and the severity of the trauma. The SGZ-DG had more FJB-positive neurons than other hippocampal subregions, regardless of the level of severity, as early as 24 h post-injury. Another study used the same marker (FJB) to assess neuronal injury after lateral fluid percussion brain injury in rats [81]. Cell loss was evident seven days post-injury in the cortex, hippocampus and thalamus. Three hours post-injury, injured neurons appeared in the hippocampus (CA1 and DG), thalamus, and vermis of the cerebellum, and the number of degenerating neurons was greatest after one and three days in the cortex and hippocampus, respectively.

The question remains as to why newborn immature neurons in the SGZ-DG are more vulnerable to TBI, and whether we can prevent it. We know that immature neurons are electrophysiologically different from their mature counterparts, that GABA A receptors are the first receptors expressed, and that during the early stages, GABAergic transmission is excitatory because of high intracellular Cl^−^ [77]. There is a possibility that the mechanism for the observed vulnerability of the hippocampus may be due to GABA-mediated excitotoxicity [82]. However, mechanisms for hippocampal neuron vulnerability are not known. ER stress has recently been assigned a role in the apoptotic death of newborn neurons after TBI through the activation of the pro-apoptotic transcription factor CHOP [83,84]. ER stress results in the accumulation of unfolded proteins, which leads to the activation of the PERK-eIF2 pathway. PERK phosphorylates eIF2, which inhibits general protein synthesis, thereby reducing ER stress. If the stress is intense and cannot be resolved by this pathway, activating transcription factor 4 (ATF4) increases the expression of CHOP, initiating apoptotic cell death. Therefore, Hood and colleagues used CHOP knockout mice to evaluate the role of this protein in TBI-induced apoptosis of doublecortin (a marker of hippocampal newborn neurons) positive neurons. They also demonstrated that the pharmacological inhibition of PERK by GSK2606414 exacerbated newborn neuron loss, and the administration of guanabenz (a drug that causes dose-dependent increases in eIF2 alpha phosphorylation) can modify the protein production rates, therefore reducing newborn neuronal loss.

## 3. Blood–Brain Barrier Disruption and Neuroinflammation

Disruption of the BBB after TBI results in hyperpermeability, increased intracranial pressure, edema, and decreased cerebral perfusion pressure, perpetuating cellular damage and hypoxia. Primary lesion leads to secondary injuries through neuroinflammation and cellular and cytotoxic edema, resulting in more neuronal death. These secondary lesions are mainly mediated by the activation of resident microglia and the recruitment of immune cells such as lymphocytes, monocytes, and neutrophils. Immunity cells will reach brain parenchyma two to 48 h after the trauma. Activation leads to increases in metalloproteinases, proteases, ROS, and reactive nitrogen species (RNS), boosting BBB and cellular damage [85,86,87,88].

The inflammatory response is initiated in resident cells, such as microglia and astrocytes, which detect pathogen-associated molecular patterns (PAMPs) and damage-associated molecular patterns (DAMPs), or by an increase of ATP that activates the mTOR pathway [89]. When exposed to an inflammatory environment, these can perpetuate the inflammatory response through differentiation to type 1 or classical activation, resulting in an enhancement of proinflammatory cytokines such as IL-6, IL-1, TNF-α and nitric oxide synthase production [90]. IL6 and TNF-α are responsible for increasing BBB permeability, IL-1 induces astrocytes to promote the expression of VCAM-1/ICAM-1 (vascular cell adhesion molecule -1 and intercellular cell adhesion molecule) in the endothelium, therefore perpetuating immune cell entry to the CNS (central nervous system), while the overstimulation of nitric oxide synthase results in more OS by increasing RNS (reactive nitrogen species) [91,92].

Microglia are CNS resident immune cells with pleiotropic functions. When exposed to a proinflammatory-enriched environment, microglia can transform into their M1 type, also known as the classical type, in order to control damage, eliminate residues, and repair tissue [93,94]. It has been shown that microglia not only mediate acute inflammatory response, but are also involved in the subsequent tissue remodeling and modulation of inflammation through M2-type differentiation. In response to inflammation, M1 microglia produce proinflammatory cytokines and microparticles, modulate the neuronal microenvironment, and, through astrocytes, can indirectly influence blood brain perfusion [95,96,97]. Chronic M1 microglial activation is related to neural degeneration due to sustained inflammatory states [98]. Therefore, the proper modulation of microglia function and switching M1 microglia to M2 can prevent the perpetuation of secondary injury-related brain damage.

Ndode-Ekane and colleagues shed some light on the complex role of T-lymphocytes (T cells) after TBI. According to their findings, and those of other authors, lymphocytes migrate soon after TBI, reaching their peak one to two days post-injury, and some remaining cell clusters are found up to 90 days post-injury [99]. The higher the presence of T cells, the poorer the neurological recovery [99,100]. T cell migration is a consequence of BBB disruption, increased endothelial expression of adhesion molecules such as VCAM/ICAM-1, excessive ROS production, and cell-mediated activation and proliferation [100,101]. CD8+ T cells sustain inflammatory states and prompt cellular damage through the activation of other cells, such as microglia, neutrophils, macrophages and astrocytes, and the production of granzyme B, which causes neuron myelin degeneration [102]. B cells have also been related to TBI-secondary injury due to autoreactive antibody production directed to brain-specific proteins freed into the medium after the trauma [102,103]. Neutrophils are also protagonists of inflammation after TBI because, like lymphocytes, they quickly migrate to the brain parenchyma through the BBB, attracted by the chemokine (C-C motif) ligand. Once in the brain parenchyma, they interact with lymphocytes and resident cells, produce cytokines and chemokines to potentialize immune reactions such as CXCL-1 and CXCL-2, release metalloproteinases, and present antigens and phagocytes in order to clean the extracellular space and prepare the tissue for scarring [85,88].

Focusing on astrocytes, this type of glia not only contributes to neuroinflammation, but also to maintaining a homeostatic balance for neurons by regulating synaptic and extrasynaptic glutamate and other neurotransmitters, stabilizing the BBB, and modulating synaptic activity, among others [104,105]. Regarding inflammation [106], once differentiated to A1 by microglia, astrocytes will quickly lose their synaptic functions and phagocytic capacity, and will start killing neurons and oligodendrocytes with no clear mechanism, but likely by inducing cellular apoptosis as the blockage of caspases 1 and 2 prevents cellular death [107]. Taking these findings together and connecting high [Ca^2+^]_i_ consequences, we address again the importance of modulating Ca^2+^ entry to cells, including astrocytes.

Regarding glutamate uptake, Dorssett and colleagues showed that the post-TBI activation of different kinases, such as kinase B (Akt) and protein kinase C (PKC), negatively modulates glutamate transporter 1 (GLT-1) function in the frontal cortex 24 h after injury [108]. GLT-1 is the most important channel of glutamate uptake in rats. In that sense, its homologous human excitatory amino acid transporter 2 (EAAT2) can also be affected after TBI [109]. In an old mouse model, Gupta and colleagues showed GLT-1 is briefly downregulated after brain trauma [110]. This means that, no matter the model or the time after trauma, glutamate reuptake is altered in astrocytes and neurons, leading to high extracellular concentrations, thus activating different glutamate receptors which promote the influx of Ca^2+^ to the cell and activating programmed cell death.

**Figure 1:**
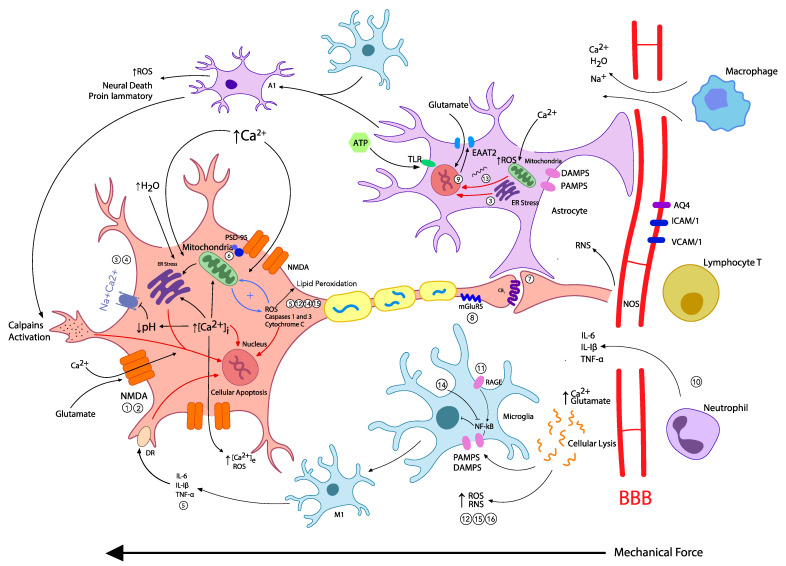
Illustration of pathophysiological events leading to excitotoxicity after TBI. BBB disruption leads to edema, changes in ionic homeostasis, and the migration of systemic immune cells. Release of intracellular contents in the medium activates microglia and astrocytes, enhancing OS, inflammation, and neural damage. Increase in extracellular [Ca^2+^] and glutamate activates NMDAr, while mechanical activation and mechanoporation of NMDAr lead to an increase in [Ca^2+^]_i_, creating an environment stressful to mitochondria, ER, and DNA, and a decrease of pH. Many pathways lead to cellular apoptosis: organelle stress and the activation of caspases, calpains, and death receptors. In black: events related to the activation and flux of ions. In red: pathways related to cellular apoptosis. Numbers address potential therapies listed in Table 1. [Ca^2+^]_i_: concentration of intracellular calcium; [Ca^2+^]_e_: concentration of extracellular calcium; BBB: blood–brain barrier; ROS: reactive oxygen species; RNS: reactive nitrogen species; NMDAr: *N*-methyl-d-Aspartate receptors; TLR: toll-like receptor; ATP: adenosine triphosphate; EAAT2: excitatory amino-acid transporter 2; NOS: nitric oxide synthase; DAMPS: damage-associated molecular patterns; PAMPS: pathogen-associated molecular patterns; AQ4: aquaporin-4; ICAM1: intercellular cell adhesion molecule-1; VCAM1: vascular cell adhesion molecule-1; mGluR5: glutamate metabotropic receptor; RAGE: receptor for advanced glycation end products; ER: endoplasmic reticulum; PSD-95: postsynaptic density protein-95; DR: death receptor.

In conclusion, enhancers and perpetuators of excitotoxicity include the BBB, glial cells and immunity cells. They are activated after trauma through different mechanisms such as ATP release to the cellular medium, cytokine production relying on the activation of NF-kB (nuclear factor kappa-light-chain-enhancer of activated B cells) triggered by DAMPS and PAMPS, and changes in cellular structure and function promoted by a proinflammatory environment. We reflect about how to modulate these enhancers later in this paper. We summarized these pathways in Figure 1.

## 4. Potential Therapies

Many drugs and compounds have emerged as potential therapies for TBI; however, few have strong clinical evidence to support their use. We analyzed the available evidence regarding therapeutic agents for excitotoxicity- and inflammation-mediated cellular damage and summarized it in Table 1.

### 4.1. NMDA Modulation

Modulation of the NMDA receptor has been widely studied in diverse pathologies. Regarding TBI, clinical evidence with classical NMDA inhibitors such as amantadine and memantine is encouraging, but confusing. Giacino and colleagues performed a multicenter randomized clinical trial in patients with severe TBI. In these patients, 200–400 mg of amantadine against placebo showed increased recovery rate after 4 weeks of administration [130]. Consistent evidence appraised the efficacy of amantadine and other NMDA antagonists with similar results [131,132,133]. Nonetheless, it must bear in mind that these studies have confounding factors such as the concomitant use of other medications, the natural progress of the disease, and the severity of head trauma. There is also an intrinsic problem in blocking NMDAr, as glutamate is an essential neurotransmitter related to memory consolidation, neuroprotection, and cell survival, and, in that sense, an effective blockade of NMDAr without altering other beneficial functions is very hard to achieve [29,134,135]. However, not all NMDA antagonists have shown clinical efficacy in TBI [136]. Other well-studied NMDAr antagonists such as MK801 [137] and dextromethorphan/quinidine [138] have been shown to reduce excitotoxicity, OS, microglial activation, and, therefore, cellular damage in animal and human studies.

Allosteric modulators have shown better pharmacological properties as they selectively block GluN2B, avoiding the undesirable effects of an NMDA blockade [34]. This prompted the study of selective GluN2B inhibitors in TBI. Taxoprodil, a GluN2B inhibitor, was studied in a randomized, double-blind, placebo-controlled study to evaluate the efficacy of a 72-h intravenous infusion in patients with severe TBI, with improved mortality and scores in the dichotomized Glasgow scale, although neither showed a significant difference [114]. Ifenprodil, another GluN2B inhibitor, has been shown to reduce excitotoxicity by modulating [Ca^2+^]_i_ concentration through blockade of NMDAr in the presence of agonists or mechanical forces [45], and has also demonstrated reduced BBB breakdown, cerebral edema, and injury volume after controlled cortical impact in rats [113].

On the other hand, there have been studies that suggest NMDA receptors have different pathophysiological implications in TBI depending on the time post-injury. Initially, there is an overactivation of these receptors, leading to excitotoxicity; however, this is followed by the desensitization and loss of functional NMDA receptors from more than one hour to seven days post-injury. This was demonstrated by quantitative autoradiography of MK801 binding to NMDAr [139]. Therefore, under this premise, a partial agonist, d-cycloserine (DCS), was evaluated in TBI. Temple and colleagues [111] evaluated DCS in a fluid percussion TBI model in rats, with a daily intraperitoneal injection of DCS (10–30 mg/kg) given from 24 h until 15 days post-injury. The spatial memory of the rats was then tested in the Morris water maze, and they found a dose-dependent improvement in the treated group. Similar studies in mice have shown improvement in neurobehavioral function at the same doses beginning at 24 h post-injury [112].

During TBI, excessive glutamate release activates glutamate receptors including mGLUR5. A study evaluated mGLUR5 brain distribution in rat cortices following TBI and subsequent administration of (R,S)-2-chloro-5-hydroxyphenylglycine (CHPG) as a selective mGluR5 agonist. Results showed that mGLUR5 was upregulated after TBI in neurons, astrocytes, and microglia. The use of delayed CHPG showed a decrease in the number of degenerating neurons following TBI due to the reduction of excitotoxic damage [120]. Recent encouraging evidence has shown mGLUR5 can modulate inflammation and neurotoxicity in a model of Parkinson’s disease, and scientists are also getting encouraging results in other fields [140,141]. We consider this body of evidence as encouraging for the prospects of mGLUR5 modulators being a feasible therapy in TBI [142,143]. Nonetheless, we do not have a complete understanding of mGLUR5 kinetics and how it can decrease neurotoxicity and neurodegeneration; therefore, more research still needs to be carried out.

### 4.2. Dantrolene

Dantrolene is a muscle relaxant that has been used as an anti-spastic agent and for the treatment of malignant hyperthermia and neuroleptic malignant syndrome, among other pathologies. Dantrolene acts as a ryanodine receptor (RyR) antagonist; said antagonism ends up being beneficial, as the blockade of the RyR reduces the calcium-induced calcium release from the endoplasmic reticulum. This mechanism has mainly been studied in skeletal muscle; however, studies in cultured neurons have shown that dantrolene can block up to 70% of the Ca^2+^ rise coming from the endoplasmic reticulum because of NMDAr activation, as well as reducing neuronal apoptosis [115]. The dose required for the treatment of malignant hyperthermia and neuroleptic malignant syndrome is similar, oscillating between 1 and 2.5 mg/kg for neuroleptic malignant syndrome and 2.5 mg/kg for malignant hyperthermia [144]. As for the dosage used to achieve the desired effect in TBI, studies have shown how an interval from 10–100 μM of dantrolene administered during the appropriate therapeutic window will target and reduce excitotoxicity. Said window appears to be 40 min either before or after the injury in in vitro models and approximately 30 min for in vivo models, making this narrow therapeutic window one of the limitations for its usage [145]. We hypothesize that reducing the Ca^2+^ efflux from the ER will reduce [Ca^2+^]_i_, thus reducing excitotoxicity [145,146,147,148,149] and positioning dantrolene as a promising therapy for patients with acute TBI; nevertheless, further clinical studies are still needed to make a definitive clinical recommendation.

### 4.3. Vitamin B12

Vitamin B12 has been studied widely in animal models for its antinociceptive properties, linking multiple mechanisms of action such as neuronal regeneration, myelin synthesis, Schwann cell differentiation, and the induction of axonal growth to this antinociceptive effect [150,151,152]. These properties motivated the study of vitamin B12 in TBI. A study was conducted to assess the effect of vitamin B12 on nerve regeneration and on ER stress after TBI in vivo and in vitro. Controlled cortical impact was performed in male mice, some of which were subsequently treated with vitamin B12 by the intraperitoneal route, and the TBI + vitamin B12 group was compared to the TBI group. The results showed that vitamin-B12-treated TBI groups had reduced ipsilateral brain edema (assessed by brain water content), better functional recovery after seven days (evaluated by Garcia test), less tissue damage in the histological morphology, reduced caspase-12-dependent apoptosis (shown by immunofluorescence staining), downregulation of the ER stress-related apoptosis signaling pathway proteins (GRP78, IRE1α, XBP-1 and CHOP) evaluated by Western blot and immunofluorescence staining, and maintained microtubule stability (a determinant of axonal growth) [116]. Therefore, vitamin B12 could have a role in preventing specific hippocampal cell loss (see Section 2.4). Even though the role of vitamin B12 in TBI is very new and not many studies are available, the studies made in animals for nociceptive, nociplastic, neuropathic pain and neuropathy [153,154], combined with the mechanisms described in this review, suggest it is possible that vitamin B12 could work as a logical, effective, and safe therapy which requires further research as a potential treatment in TBI to mitigate secondary injuries and, therefore, long-term disability.

### 4.4. Ceftriaxone

This beta-lactam antibiotic is proposed as a possible treatment for TBI. Ceftriaxone penetrates the BBB and increases the expression of EAAT2. As previously discussed, glutamate is the principal contributor for excitotoxicity and its consequences. Glutamate transporters, such as GLT-1/EAAT2, have been shown to be reduced 24 h following TBI, thus reducing glutamate reuptake and contributing to excitotoxicity. The potential therapeutic capacity of ceftriaxone in TBI is thought to be because it can activate the EAAT2 promoter in human fetal astrocytes [155,156]. In a rat model of subarachnoid hemorrhage (SAH), the use of ceftriaxone was evaluated to prevent early brain injury. Intracisternal treatment with ceftriaxone led to improved neurological outcomes and it was able to lessen glutamate accumulation after SAH [121]. Furthermore, a recent study evaluating ceftriaxone in a TBI rat model found that the ceftriaxone-treated group had significantly lower intracranial pressure, decreased infarct volume, decreased neuronal apoptosis, increased GLT-1 expression in both neurons and microglia, and improved motor dysfunction [157].

### 4.5. Minocycline

Tetracyclines have been studied beyond their antibiotic properties, and, as a result, they have been linked to neuroprotection, as well as anti-inflammatory and antiapoptotic activity. Minocycline is a lipid-soluble, second-generation tetracycline that has potential benefits in TBI. A study conducted to evaluate if minocycline reduced excitotoxicity in primary neuronal cultures showed that it increased neuronal survival [158]. The study showed minocycline prevented excitotoxic-induced microglial proliferation, and excessive release of nitric oxide (NO) and IL-1β. In addition, it has been shown to reduce caspase-1 activation following TBI in mice, as has been seen before in many animal models of ischemia and Huntington’s disease. However, the exact mechanism by which minocycline inhibits caspase 1 is still to be elucidated and it is beyond the scope of this review [159]. Furthermore, a study evaluating the effects of minocycline in TBI-induced neurological impairment found that minocycline increased neuronal viability, caused iron chelation in vitro, and attenuated neurological impairment in rats [117]. Minocycline safety has been studied in a phase IIa clinical trial in TBI (NCT01058395). The study consisted of administering intravenous minocycline via a central line within six hours of the initial injury to patients with a GCS of less than 12, and this treatment continued for seven days. It was found to be safe for moderate to severe TBI [160].

### 4.6. PSD-95 Inhibitors

NMDA and neuronal NOS are linked via the post synaptic density protein (PSD-95), a guanylate kinase. This protein binds the GluN2B subunit of NMDA to the amino terminus of nNOS, finally creating the NMDA/PSD-95/nNOS complex. Thus, NMDA overstimulation in excitotoxicity leads to neurotoxic levels of NO, which participates in cytotoxic actions, ultimately leading to neuronal death [161]. Consequently, the inhibition of PSD-95 has emerged as a possible therapeutic target. A recent study evaluated an inhibitor of this PSD-95/NMDA interaction called ZL006, a small molecule that can readily cross the BBB and that had good outcomes in mouse stroke models [118,162]. This study evaluated ZL006 in cortical neuronal cultures, showing reduced glutamate-induced neuronal death, and showed improvement in somatosensory, motor, and memory deficits, as well as cognitive impairment in rodents [118]. Furthermore, UCCB01-147, a dimeric PSD-95 inhibitor, was evaluated in rats to assess its neuroprotective effects. Rats were subjected to controlled cortical impact followed by 10 mg/kg of UCCB01. However, it failed to reduce cell death [163].

### 4.7. MicroRNA (miRNA), Mesenchymal Stem Cells (MSC’s), and Exosome Therapy

MiRNA are a family of short, non-coding RNA molecules that can regulate gene expression at the post-transcriptional level [164]. They have been implicated in the pathophysiology of cancer, as well as cardiovascular and metabolic diseases. They can regulate gene expression through the degradation of mRNA or by inhibiting its translation [164]. In a TBI rat model, the upregulation of miRNA-21 was found to inhibit apoptosis and promote angiogenesis [165]. Following controlled cortical impact in rats, the upregulation of miRNA-9-5 improved cellular viability and improved apoptosis by the post-transcriptional modulation of Ptch-1 (patched protein 1). It also promoted the expression of factors that promote angiogenesis (VEGF, MMP-9 and cyclin D1) [166].

The important role of miRNA has also been elucidated when studying the use of mesenchymal stem cells (MSCs) in TBI. Exosomes (membrane vesicles) are thought to be an important part in the therapeutic capacity of MSCs because they can carry proteins, lipids, mRNA, and miRNA that can be transferred among cells. Cell-free exosomes derived from MSCs are therefore considered a potential therapeutic strategy, as they can deliver specific miRNA with neuroprotective effects. Xin and colleagues found that rats exposed to treatment with MSCs after middle cerebral artery occlusion significantly increased the expression of miRNA-133b, which contributed to neurite outgrowth [167,168]. Zhang and colleagues studied the systemic administration of cell-free exosomes extracted from human bone marrow MSCs in rats after TBI [125]. Rats were subjected to controlled cortical impact, and a venous injection of exosomes was administered 24 h later. The exosome treatment group did not have a reduction in lesion size, but had sensorimotor functional recovery and improved spatial learning by promoting angiogenesis and neurogenesis and reducing neuroinflammation [125].

### 4.8. Progesterone

Progesterone is a neurosteroid that is involved in neuroprotection and in mechanisms regarding repair after brain injury [169]. It has been tested in various injury models, including TBI, spinal cord injury, and stroke. Some clinical trials have also been carried out, with reductions in mortality and improved neurologic outcomes [170], whereafter a randomized, double-blind, placebo-controlled clinical trial was held. This study analyzed one hundred trauma patients with a GCS of 4–12, showing no serious adverse effects, and reduced mortality in the progesterone group [126]. Various mechanisms have been proposed for these neuroprotective effects. Progesterone influences the expression of genes involved in the regulation of the inflammatory response, as well as apoptosis. It also reduces edema after TBI by modulating AQ4 (aquaporin 4) expression on astrocytes [171]. There are also studies showing that progesterone can reduce lipid peroxidation by inhibiting free radical formation [169]. Regarding excitotoxicity, progesterone can attenuate neuronal excitotoxicity by inhibiting voltage-gated calcium channels [172]. However, in 2014, a phase III randomized, placebo-controlled clinical trial was held to evaluate the efficacy of intravenous progesterone in the treatment of non-penetrating TBI. The study analyzed 882 patients with a GCS of 4–12. Nevertheless, after the second interim analysis, the trial was stopped because of futility. There were no significant differences in mortality between groups [127]. Thus, progesterone’s role as neurosteroid is still secondary, despite its importance.

### 4.9. Endocannabinoids

Recent published data shows cannabis can have a use in TBI [173]. When 2-arachidonylglycerol (2-AG) was administered to mice after moderate or severe TBI, activation of cannabinoid receptor type 1 (CB1) attenuated the inflammatory response, protected the BBB, and improved clinical recovery. Similar results were obtained by Tchantchou et al. [119] using WWL70 which acts as an inhibitor of monoacylglycerol lipase, an enzyme responsible for degradation of 2-AG, the most abundant endocannabinoid. WWL70, as well as other selective inhibitors of endocannabinoid metabolism such as URB597 [174] palmitylsulfonyl fluoride (AM374) [175] have given similar results. This shows the endocannabinoid system has potential therapeutical effects by modulating inflammation, and even excitotoxicity, in TBI. It is important to recognize that the setting in which CB1 is activated can have different consequences, as some studies suggest that CB1 agonism can act as an enhancer of oxidative stress and inflammation [176]. However, no human studies in patients with acute TBI in the setting of neuro-ICU care have been done yet.

### 4.10. Intestinal Microbiota

Microbiota’s role on human physiology is an emerging field of study. Many authors have pinpointed bacteria as one of the main actors in the metabolism of nutrients, regulation of the immune system, and activation of gene expression, among others, while cooperating and cohabitating with human beings [177]. It is worth saying that, by this point, there is no such thing as a “bad” microbiota and authors have addressed a lack of microbiota diversity or aggressive species colonization as an altered microbiota. Taking this into account, one must have in mind that intestinal microbiota varies depending on the individual, their diet habits, and specific situations such as antibiotic use. Gut–brain signaling is a fact, and the vagus nerve, along with the immune and vascular systems, is the bridge in between the two. Bidirectional communication and compliance can be, and has been, used as pharmacological targets in different conditions. For example, in autism, antibiotic therapy, probiotics, and prebiotics have shown improvement in patients’ symptomatology by altering host microbiota [178]. The hypothesis argues that these treatments enhance fatty acid metabolism, thereby improving the supply of the main products for brain function and regulating systemic inflammation by controlling proinflammatory cytokines. Common molecular pathways, such as pyrin domain-containing 3 (NLRP3) activation, are known to alter intestinal cell homeostasis, thereby promoting bacterial inflammatory states [179,180]. NLRP3 inflammasome is also activated after TBI, ultimately leading to calpain activation through the rise of [Ca^2+^]_i_.

TBI patients can have comorbidities such as obesity and diabetes, which are well known to induce systemic inflammatory states [181]. Specific prebiotics enhance anti-inflammatory microorganisms, such as *F. prausnitzii*, that, through the augmentation of short chain fatty acid production, can regulate systemic inflammation and can therefore modulate brain parenchyma inflammation [182,183]. Nonetheless, there is still much more research to be done in this field [184], as TBI patients frequently have concomitant infections which are treated with antibiotics, which can then influence microbiota diversity and function. We consider that encouraging evidence in autism can be applied in TBI [122]. A directed antibiotic regimen depending on individual microbiota characterization and a consequent reconstitution through microbiota transfer therapy enhancing, for example, the growth of *Acinetobacter* spp., *Bacteroides fragilis*., and *Proteobacteria* [185], etc., as well as diet, probiotics and prebiotics, can modulate inflammation and oxidative stress, attenuating disability and prompting recovery after TBI.

### 4.11. RAGE Inhibitors

Inflammation following TBI can activate the receptor for advanced glycation end products (RAGE). RAGE activates NF-kB [186], and researchers have broadly addressed an increase of NF-kB activation after TBI [187,188]. Recently published data showed an increased expression of high mobility group box protein 1 (HMGB1), which activates inflammation in the acute setting, but has a subsequent correlation to neural genesis by targeting specific gene expression [123,189,190]. HMGB1–RAGE interaction is well known, but not clearly understood. Apparently, RAGE inhibits HMGB1 expression by direct binding and through NF-kB-mediated endocytosis. Given this complex interaction, we consider the inhibition of RAGE, but not HMGB1, as a therapeutic target to promote long-term neurogenesis and as an attenuator of inflammation. Nonetheless, no studies have been completed with RAGE inhibitors following TBI; despite that, we consider that the effects of RAGE inhibitors in other fields can shed some light on how we could start exploring this receptor in TBI [191,192].

### 4.12. Zinc Supplementation

Excess free zinc was initially thought to be part of the secondary injury mechanisms following TBI. Therefore, zinc chelation was evaluated for a while as a potential therapy [193,194,195]. However, the chelation of zinc was subsequently associated with neuronal damage due to overexcitation [196] and increased apoptosis and necrosis [197]. Moreover, it has been shown that patients with severe head trauma have urinary zinc excretion up to 14-fold higher than normal [198]. Zinc deficiency impairs neurogenesis [199], cell proliferation, neuronal survival, and contributes to the inflammatory response [200]. Furthermore, zinc can function as a neurotransmitter or secondary messenger, and it also regulates cellular oxidant production and signaling cascades in the brain [200]. Therefore, zinc supplementation, rather than zinc chelation, was evaluated in a randomized, controlled clinical trial in 100 patients with severe head trauma, aged 18–65 years old, with a GCS of 6–8. Patients were treated with 528 mg of zinc sulfate for 15 days; results showed improvement of the inflammatory markers, SOFA and GCS, although mortality did not significantly differ between groups [124].

### 4.13. Antioxidants

Free radical scavengers such as glutathione and edaravone are reasonable therapies, as they are antioxidants that can donate electrons to neutralize the toxic effects of ROS [54,201]. In a weight drop model conducted by Wang and colleagues, male rats with induced brain trauma in the right cerebral cortex were subsequently treated with edaravone two hours and 12 h after injury. Edaravone significantly reduced hippocampal neuronal loss, oxidative stress, BBB permeability, and neurological deficit after recovery. Therefore, edaravone might be considered neuroprotective in the case of stroke and TBI [128,202].

### 4.14. Curcumin

Curcumin (Cur) is a natural polyphenol found in the plant *Curcuma longa* (turmeric) [203]. It has been explored for its properties in promoting neurogenesis and improving memory, as well as for its anti-inflammatory functions. Sun and colleagues evaluated Cur in a murine model of TBI, in which they administered 30 mg/kg of Cur vs. vehicle treatment to rats, and subsequently evaluated neuroinflammation (by assessing inflammatory factors, astrocyte hypertrophy, and activated microglia in the hippocampus), and performed behavioral water maze studies. The authors found Cur ameliorated TBI-impaired spatial memory, reduced chronic neuroinflammation, reduced inflammatory factors, and increased neurogenesis in the hippocampus [129]. The mechanism by which Cur can cause these effects is thought to be through increased signaling of the BDNF/TrkB/PI3K/Akt pathway. The BDNF and TrkB pathways play a role in reducing inflammation and promoting hippocampal neurogenesis, while PI3K/Akt signaling has been linked with the promotion of neuronal survival and reducing neuroinflammation [204]. Furthermore, the neuroprotective effects of Cur have been attributed to the activation of the Nrf2-ARE (nuclear erythroid 2-related factor 2- antioxidant response element) pathway, which modulates oxidative stress. This was documented in a weight drop TBI model in rats in which Cur was shown to increase the translocation of Nrf2 from the cytoplasm to the nucleus [205].

## 5. Conclusions

In conclusion, traumatic brain injury represents a high burden for the patient and their family, as well as for the healthcare system. Damage to the brain following TBI can be divided into primary and secondary injuries, the latter being the focus of this review. Managing the secondary mechanisms that lead to tissue damage following TBI has been a major challenge as these processes are related to tissue recovery, scar formation, and residue metabolism, leading to further tissue rehabilitation. Despite having many potential drugs, it is difficult to assess which of them can have a positive impact in the recovery pathways. Given the many mechanisms involved, a vast number of potential compounds, most coming from drug repurposing, have been proposed as potential therapies. Treatments ranging from drugs targeting different subunits or associated proteins of the NMDA receptor to antibiotics, progesterone, vitamin B12, CB1 agonism, prebiotics, and even mesenchymal stem cell therapy have shown a potential therapeutic role in TBI. However, few have achieved the bench to bedside translation. We strongly believe that widely studied compounds, such as selective GluN2B inhibitors and vitamin B12, could have a relevant role in the treatment of TBI and can have a positive impact on long term disability, thereby reducing disease burden and costs to the health care system.

## Figures and Tables

**Table 1:** Summary of preclinical and clinical evidence of potential therapies for TBI.

Novel Potential Therapies	Mechanism of Action	Pre/Clinical Evidence	Result	Reference
1. NMDAr partial agonists (d-cycloserine)	Partial agonism of NMDA receptors in the loss of function period following TBI	Lateral fluid percussion TBI model. Following TBI, rats were injected with daily intraperitoneal d-cycloserine (10–30 mg/kg), which was given from 24 h until 15 days post injury. A control group received vehicle. (Animal study)	30 mg/kg dose significantly reduced memory deficits compared to the control group. 10 mg/kg was ineffective in attenuating memory deficits. Loss of function of NMDA receptors was >1 h—7 days post injury	[111]
	Partial agonism of NMDA receptors in the loss of function period following TBI	Weight-drop TBI model in male mice, subsequently treated with 10 mg/kg of d-cycloserine (i.p.) or vehicle in different regimens. (Animal study)	Functional recovery assessed by NSS score was better in the d-cycloserine treated group at 24 or 72 h post trauma	[112]
2. NMDAr subunit GluN2B antagonists (Ifenprodil, Taxoprodil)	Ifenprodil: selective GluN2B inhibitor	Controlled cortical impact TBI model in rats. Infenprodil vs. saline was injected by the intraperitoneal route immediately after injury, and then every 90 min until 6 h after injury. (Animal study)	BBB breakdown, brain edema and injury volume were lower in the ifenprodil-treated group vs. the saline-treated group.	[113]
	Taxoprodil: selective GluN2B inhibitor	A randomized, double-blind, placebo-controlled study to evaluate the efficacy of a 72-h intravenous infusion in patients with severe TBI. 404 males and females were treated within 8 h of injury. (Human study)	Taxoprodil-treated group had better outcome con the Glasgow outcome scale at 6 months. Mortality rate was 7% less than in the placebo group.	[114]
3. Ryanodine receptor antagonist (Dantrolene)	Ryanodine receptor antagonist, blockage of calcium induced calcium-release from the ER, protecting against glutamate induced excitotoxicity	Cerebral cortical neurons of mice were exposed to KCl or glutamate alone or in combination with dantrolene. (Animal study)	Dantrolene reduced the glutamate induced increase in [Ca^2+^]_i_ by 70%	[115]
4. Vitamin B12	↓ ER stress related apoptosis	Controlled cortical impact was performed in male mice who were subsequently treated with vitamin B12 by the intraperitoneal route. The TBI + vitamin B12 group was compared to the TBI group. (Animal study)	TBI + VB12 showed: ↓ ipsilateral brain edema, tissue damage ↓ GRP78, IRE1α, XBP-1 and CHOP Better functional recovery after 7 days	[116]
5. Protein synthesis inhibitors (Minocycline)	↓ excessive release of NO, ↓ Activation of Caspase 1 and 3, Fe^2+^ and Fe^3+^ chelating activity, ↓ IL-1B	Rats were subjected to weight drop model of TBI, and were subsequently divided intro TBI + vehicle, or TBI + minocycline at different doses. (Animal study)	Minocycline treated groups had increased neuronal viability, chelating activity for iron in vitro, and attenuated neurological impairment	[117]
6. PSD-95 inhibitors (ZL006)	Inhibition of PSD-95 reducing neurotoxic levels of NO	Female mice were subjected to controlled cortical impact, after 30 min post injury they were treated with ZL006 or vehicle. (Animal study)	ZL006 (PSD-95 inhibitor) treated group had reduced expression of apoptotic markers, improved neuroscores, and attenuated cognitive impairment	[118]
7. Endocannabinoids	2-arachidonylglycerol attenuates inflammatory response, protects BBB	Male mice were subjected to controlled cortical impact. 30 min following TBI, WWL70 (inhibitor of the principal enzyme that degrades 2-AG) was administered vs. saline. (Animal study)	Improved motor coordination and working memory performance, reduced lesion size in the cortex and neurodegeneration in the dentate gyrus	[119]
8. mGLU5 agonists (CHPG)	Reduction of excitotoxic damage	Male rats were subjected to a weight drop TBI model and were distributed among sham, TBI, TBI + vehicle, and TBI + CHPG (mGluR5 agonist) groups. (Animal study)	The use of delayed CHPG led to a decrease in the number of degenerating neurons. Reduced excitotoxic damage	[120]
9. Bacterial cell wall synthesis inhibitors (Ceftriaxone)	Activates the EAAT2 promoter in human fetal astrocytes, increasing glutamate reuptake	Rat model of SAH compared SAH + intracisternal treatment with ceftriaxone at different doses compared to SAH + saline. (Animal study)	Decreased hippocampal neuronal apoptosis, improved neurological outcomes and reduced extracellular glutamate concentration	[121]
10. Prebiotics	Regulation of systemic inflammation	Open-label trial of MTT in 18 participants with autism. (Human study)	GI symptom relief, autism severity was lower (according to CARS)	[122]
11. RAGE inhibitors (Glycyrrhizin, FPS-ZM1)	Rage inhibition leads to ↑ HMGB1 which activated neural genesis, attenuation of glycosylation, antioxidative stress, metal ion chelation, and reduced scavenging of reactive 1,2-dicarbonyl compounds or ROS/RNS	Glycyrrhizin (10 mg/kg) and FPS-ZM1 (1 mg/kg) were administered to inhibit microglial RAGE and HMGB1 respectively (Animal study)	Blockade of RAGE/HMGB1 suppresses proinflammatory microglia polarization and granted neuroprotection	[123]
12. Zinc	Regulates cellular oxidant production and signaling cascades in the brain, modulates hippocampal neurogenesis	Double-blind randomized placebo-controlled clinical trial evaluated 100 patients with severe head trauma. Patients received placebo vs. 120 mg Zinc (Human study)	Improvement in the SOFA, Glasgow coma scale, and inflammation factors	[124]
13. MicroRNA/exosome therapy	Regulate gene expression by degradation of mRNA or by inhibiting its translation	Rats were subjected to controlled cortical impact; 24 h later, exosomes were injected intravenously. (Animal study)	Sensorimotor functional recovery, improved spatial learning by promoting angiogenesis, neurogenesis and reducing neuroinflammation	[125]
14. Neurosteroids (Progesterone)	Modulates inflammatory response, apoptosis and AQ4, regulation of inflammatory response	Randomized double-blind, placebo-controlled clinical trial was held. One hundred trauma patients with a Glasgow score of 4–12 were analyzed, and the subjects were randomized to receive intravenous progesterone or placebo. (Human study)	Lower 30-day mortality rate than controls	[126]
		Randomized double-blind, placebo-controlled clinical trial evaluated 882 patients with non-penetrating TBI. Progesterone was administered i.v. within 4 h of trauma and compared with placebo. (Human study)	No mortality difference was seen between the two groups. The trial was stopped early because of futility.	[127]
15. ROS scavenger (Edaravone)	Donates electrons to neutralize ROS	Rats were subjected to a weight drop model and were subsequently treated with edaravone 2 h and 12 h after injury. (Animal study)	Edaravone significantly reduced hippocampal neuronal loss, reduced oxidative stress, BBB permeability and neurological deficit after recovery	[128]
16. Curcumin	↑ BDNF/TrkB/PI3K/Akt signaling	Rats were subjected to a TBI model and 28 days after were treated with 30 mg /kg of Cur vs. vehicle. (Animal study)	Cur ameliorated TBI-impaired spatial memory, reduced chronic neuroinflammation, reduced inflammatory factors and increased neurogenesis in the hippocampus	[129]

**KCl**: potassium chloride; **ER**: endoplasmic reticulum; **GRP78**: glucose regulated protein 78,000; **IRE1α**: inositol-requiring kinase/endonuclease 1; **XBP-1**: X-box binding protein 1; **CHOP**: C/EBP homologous protein; **CARS**: childhood autism rating scale; **2-AG**: 2-Arachidonoylglycerol; **EAAT2**: excitatory amino acid transporter 2; **SAH**: subarachnoid hemorrhage; **MTT**: microbiota transfer therapy; **RAGE**: receptor for advanced glycation end products; **HMGB1**: high-mobility group protein 1; **SOFA**: sequential organ failure assessment score; **mRNA**: messenger RNA; **AQ4**: aquaporin-4; **DCS**: d-cycloserine; RyR: ryanodine receptor; **GCS**: Glasgow coma scale; **miRNA**: microRNA; **MSCs**: mesenchymal stem cells; **Ptch-1**: parched protein 1; **CB1**: cannabinoid receptor type 1; **CHPG**: (R,S)-2-chloro-5- hydroxyphenylglycine; **BDNF**: brain-derived neurotrophic factor; **TrkB**: tropomyosin receptor kinase B; **PI3K**: phosphatidylinositol 3-kinases; **Akt**: protein kinase B.

## Abbreviation

TBI: Traumatic Brain Injury; NAC: North American Countries; BBB: Blood Brain Barrier; NMDAr: N-methyl D- aspartate receptor; GABA: Gamma-Aminobutyric acid; EC50: Half maximal effective concentration; AMPA: α-amino-3-hydroxy-5-methyl-4-isoxazolepropionic acid; NMDA: N-methyl D- aspartate; NOS: Nitric Oxide Synthase; ROS: Reactive Oxygen Species; ATP: Adenosine Triphosphate; DNA: Deoxyribonucleic acid; STEP: Striatal-Enriched Protein Tyrosine Phosphatase; ER: Endoplasmic Reticulum; UPR: Unfolded Protein Response; PERK: PKR-like ER kinase; ATF6: Activating Transcription Factor 6; IRE-1: Inositol Requiring Enzyme 1; GRP78/BIP: Glucose-Regulated Protein/Binding Immunoglobulin Protein; eIF2α: PERK phosphorylates eukaryotic initiation factor 2α; CHOP: C/EBP Homologous Protein; SGZ-DG: Subgranular Zone of the Dentate Gyrus; FJP: Ionic Fluorescein; ATF4: Activating Transcription Factor 4; RNS: Reactive Nitrogen Species; PAMPs: Pathogen-Associated Molecular Patterns; DAMPs: Damage-Associated Molecular Patterns; mTOR: Mechanistic Target Of Rapamycin Kinase; VCAM-1: Vascular Cell Adhesion Molecule-1; ICAM-1: Intercellular Cell Adhesion Molecule-1; CNS: Central Nervous System; PKC: Protein kinase C; AKT: Protein kinase B (PKB); GLT-1: Glutamate transporter 1; EAAT2: Excitatory amino acid transporter 2; NF-kB: Nuclear factor kappa-light-chain-enhancer of activated B cells; KCL: Potassium Chloride; GRP78: Glucose-regulated protein 78; XBP-1: X-box binding protein 1; NO: Nitric Oxide; PSD-95: Postsynaptic density protein 95; 2-AG: 2-Arachidonoylglycerol; CHPG: (R,S)-2-chloro-5- hydroxyphenylglycine; SAH: Subarachnoid Hemorrhage; MTT: Microbiota Transfer Therapy; RAGE: Receptor For Advanced Glycation End Products; HMGB1: High-Mobility group protein 1; SOFA: Sequential Organ Failure Assessment Score; mRNA: Messenger RNA; AQ4: Aquaporin-4; DCS: D-cycloserine; RyR: Ryanodine Receptor; GCS: Glasgow Coma Scale; miRNA: MicroRNA; MSCs: Mesenchymal Stem Cells; Ptch-1: Parched Protein 1; CB1: Cannabinoid Receptor type 1; AM 374: Palmitoyl Sulfonyl Fluoride; NLRP3: Pyrin Domain-Containing 3; BDNF/TrkB/PI3K/Akt: Brain-derived Neurotrophic Factor/Tropomyosin receptor kinase B/Phosphatidylinositol 3-kinase/Protein kinase B; Nrf2-ARE: Nuclear factor erythroid 2-related factor 2-Antioxidant Response Element.

## References

[1] Capizzi A., Woo J., Verduzco-Gutierrez M. (2020). Traumatic Brain Injury: An Overview of Epidemiology, Pathophysiology, and Medical Management. Med. Clin. N. Am..

[2] Hackenberg K., Unterberg A. (2016). [Traumatic Brain Injury]. Der Nervenarzt.

[3] Saban K., Griffin J., Urban A., Janusek M., Pape T., Collins E. (2016). Perceived Health, Caregiver Burden, and Quality of Life in Women Partners Providing Care to Veterans with Traumatic Brain Injury. J. Rehabil. Res. Dev..

[4] Kanmani T., Thimmappur R., Birudu R., Reddy N K., Raj P. (2019). Burden and Psychological Distress of Intensive Care Unit Caregivers of Traumatic Brain Injury Patients. Indian J. Crit. Care Med. Peer-Rev. Off. Publ. Indian Soc. Crit. Care Med..

[5] Dewan M., Rattani A., Gupta S., Baticulon R., Hung Y., Punchak M., Agrawal A., Adeleye A., Shrime M., Rubiano A. (2018). Estimating the Global Incidence of Traumatic Brain Injury. J. Neurosurg..

[6] Ma V., Chan L., Carruthers K. (2014). Incidence, Prevalence, Costs, and Impact on Disability of Common Conditions Requiring Rehabilitation in the United States: Stroke, Spinal Cord Injury, Traumatic Brain Injury, Multiple Sclerosis, Osteoarthritis, Rheumatoid Arthritis, Limb Loss, and Back Pain. Arch. Phys. Med. Rehabil..

[7] Langlois J., Rutland-Brown W., Wald M. (2006). The Epidemiology and Impact of Traumatic Brain Injury: A Brief Overview. J. Head Trauma Rehabil..

[8] Feigin V., Vos T., Alahdab F., Amit A., Bärnighausen T., Beghi E., Beheshti M., Chavan P., Criqui M., Desai R. (2021). Burden of Neurological Disorders Across the US From 1990-2017: A Global Burden of Disease Study. JAMA Neurol..

[9] Mckee A., Daneshvar D. (2015). The Neuropathology of Traumatic Brain Injury. Handb. Clin. Neurol..

[10] Davanzo J., Sieg E., Timmons S. (2017). Management of Traumatic Brain Injury. Surg. Clin. N. Am..

[11] Greve M.W., Zink B.J. (2009). Pathophysiology of Traumatic Brain Injury. Mt. Sinai J. Med. A J. Transl. Pers. Med..

[12] Pun P.B.L., Lu J., Moochhala S. (2009). Involvement of ROS in BBB Dysfunction. Free Radic. Res..

[13] Alves J. (2014). Blood-Brain Barrier and Traumatic Brain Injury. J. Neurosci. Res..

[14] Alam A., Thelin E.P., Tajsic T., Khan D.Z., Khellaf A., Patani R., Helmy A. (2020). Cellular Infiltration in Traumatic Brain Injury. J. Neuroinflamm..

[15] Winkler E., Minter D., Yue J., Manley G. (2016). Cerebral Edema in Traumatic Brain Injury: Pathophysiology and Prospective Therapeutic Targets. Neurosurg. Clin. N. Am..

[16] Jarrahi A., Braun M., Ahluwalia M., Gupta R., Wilson M., Munie S., Ahluwalia P., Vender J., Vale F., Dhandapani K. (2020). Revisiting Traumatic Brain Injury: From Molecular Mechanisms to Therapeutic Interventions. Biomedicines.

[17] Clark D.P.Q., Perreau V.M., Shultz S.R., Brady R.D., Lei E., Dixit S., Taylor J.M., Beart P.M., Boon W.C. (2019). Inflammation in Traumatic Brain Injury: Roles for Toxic A1 Astrocytes and Microglial–Astrocytic Crosstalk. Neurochem. Res..

[18] Loane D., Kumar A. (2016). Microglia in the TBI Brain: The Good, the Bad, and the Dysregulated. Exp. Neurol..

[19] Galgano M., Toshkezi G., Qiu X., Russell T., Chin L., Zhao L. (2017). Traumatic Brain Injury: Current Treatment Strategies and Future Endeavors. Cell Transplant..

[20] Sveen U., Roe C., Sigurdardorrit S., Skandsen T., Andelic N., Manskow U., Berntsen S., Soberg H., Anke A. (2016). Rehabilitation pathways and functional independence one year after severe traumatic brain injury. Eur. J. Phys. Rehabil. Med..

[21] Kreitzer N., Rath K., Kurowski B., Bakas T., Hart K., Lindsell C., Adeoye O. (2019). Rehabilitation Practices in Patients With Moderate and Severe Traumatic Brain Injury. J. Head Trauma Rehabil..

[22] Selassie A., Zaloshnja E., Langlois J., Miller T., Jones P., Steiner C. (2008). Incidence of Long-Term Disability Following Traumatic Brain Injury Hospitalization, United States, 2003. J. Head Trauma Rehabil..

[23] Zaloshnja E., Miller T., Langlois J., Selassie A. (2008). Prevalence of Long-Term Disability from Traumatic Brain Injury in the Civilian Population of the United States, 2005. J. Head Trauma Rehabil..

[24] Rigotto G., Zentilin L., Pozzan T., Basso E. (2021). Effects of Mild Excitotoxic Stimulus on Mitochondria Ca^2+^ Handling in Hippocampal Cultures of a Mouse Model of Alzheimer’s Disease. Cells.

[25] Jankovic M., Novakovic I., Dawod P.G.A., Dawod A.G.A., Drinic A., Motaleb F.I.A., Ducic S., Nikolic D. (2021). Current Concepts on Genetic Aspects of Mitochondrial Dysfunction in Amyotrophic Lateral Sclerosis. Int. J. Mol. Sci..

[26] Villanueva J.R., Esteban J.M., Villanueva L.J.R. (2020). Retinal Cell Protection in Ocular Excitotoxicity Diseases. Possible Alternatives Offered by Microparticulate Drug Delivery Systems and Future Prospects. Pharmaceutics.

[27] Kostic M., Zivkovic N., Stojanovic I. (2013). Multiple Sclerosis and Glutamate Excitotoxicity. Rev. Neurosci..

[28] Hynd M., Scott H., Dodd P. (2004). Glutamate-Mediated Excitotoxicity and Neurodegeneration in Alzheimer’s Disease. Neurochem. Int..

[29] Ng S.Y., Lee A.Y.W. (2019). Traumatic Brain Injuries: Pathophysiology and Potential Therapeutic Targets. Front. Cell. Neurosci..

[30] Kyyriäinen J., Kajevu N., Bañuelos I., Lara L., Lipponen A., Balosso S., Hämäläinen E., das Gupta S., Puhakka N., Natunen T. (2021). Targeting Oxidative Stress with Antioxidant Duotherapy after Experimental Traumatic Brain Injury. Int. J. Mol. Sci..

[31] Hansen K.B., Yi F., Perszyk R.E., Furukawa H., Wollmuth L.P., Gibb A.J., Traynelis S.F. (2018). Structure, Function, and Allosteric Modulation of NMDA Receptors. J. Gen. Physiol..

[32] Guo H., Camargo L.M., Yeboah F., Digan M.E., Niu H., Pan Y., Reiling S., Soler-Llavina G., Weihofen W.A., Wang H.-R. (2017). A NMDA-Receptor Calcium Influx Assay Sensitive to Stimulation by Glutamate and Glycine/D-Serine. Sci. Rep..

[33] Samson A., Robertson G., Zagnoni M., Connolly C. (2016). Neuronal Networks Provide Rapid Neuroprotection against Spreading Toxicity. Sci. Rep..

[34] Zhu S., Paoletti P. (2015). Allosteric Modulators of NMDA Receptors: Multiple Sites and Mechanisms. Curr. Opin. Pharmacol..

[35] Chiang V.S.-C., Park J.H. (2020). Glutamate in Male and Female Sexual Behavior: Receptors, Transporters, and Steroid Independence. Front. Behav. Neurosci..

[36] Palacios-Filardo J., Mellor J. (2019). Neuromodulation of Hippocampal Long-Term Synaptic Plasticity. Curr. Opin. Neurobiol..

[37] Ladak A., Enam S., Ibrahim M. (2019). A Review of the Molecular Mechanisms of Traumatic Brain Injury. World Neurosurg..

[38] Hardingham G.E., Fukunaga Y., Bading H. (2002). Extrasynaptic NMDARs Oppose Synaptic NMDARs by Triggering CREB Shut-off and Cell Death Pathways. Nat. Neurosci..

[39] Kunz A., Dirnagl U., Mergenthaler P. (2010). Acute Pathophysiological Processes after Ischaemic and Traumatic Brain Injury. Best Pract. Res. Clin. Anaesthesiol..

[40] Guerriero R., Giza C., Rotenberg A. (2015). Glutamate and GABA Imbalance Following Traumatic Brain Injury. Curr. Neurol. Neurosci. Rep..

[41] Tasker R. (2012). Spreading Depolarisations and Traumatic Brain Injury: Time Course and Mechanisms. Lancet. Neurol..

[42] Lee M.C., Ting K.K., Adams S., Brew B.J., Chung R., Guillemin G.J. (2010). Characterisation of the Expression of NMDA Receptors in Human Astrocytes. PLoS ONE.

[43] Kloda A., Lua L., Hall R., Adams D.J., Martinac B. (2007). Liposome Reconstitution and Modulation of Recombinant N-Methyl-d-Aspartate Receptor Channels by Membrane Stretch. Proc. Natl. Acad. Sci. USA.

[44] Maneshi M.M., Maki B., Gnanasambandam R., Belin S., Popescu G.K., Sachs F., Hua S.Z. (2017). Mechanical Stress Activates NMDA Receptors in the Absence of Agonists. Sci. Rep..

[45] Singh P., Doshi S., Spaethling J., Hockenberry A., Patel T., Geddes-Klein D., Lynch D., Meaney D. (2012). N-Methyl-D-Aspartate Receptor Mechanosensitivity Is Governed by C Terminus of NR2B Subunit. J. Biol. Chem..

[46] Hånell A., Rostami E. (2020). How Can a Punch Knock You Out?. Front. Neurol..

[47] Pettus E., Povlishock J. (1996). Characterization of a Distinct Set of Intra-Axonal Ultrastructural Changes Associated with Traumatically Induced Alteration in Axolemmal Permeability. Brain Res..

[48] Farkas O., Lifshitz J., Povlishock J.T. (2006). Mechanoporation Induced by Diffuse Traumatic Brain Injury: An Irreversible or Reversible Response to Injury?. J. Neurosci..

[49] Boothe D.L., Yu A.B., Kudela P., Anderson W.S., Vettel J.M., Franaszczuk P.J. (2017). Impact of Neuronal Membrane Damage on the Local Field Potential in a Large-Scale Simulation of Cerebral Cortex. Front. Neurol..

[50] LaPlaca M., Lessing M., Prado G., Zhou R., Tate C., Geddes-Klein D., Meaney D., Zhang L. (2019). Mechanoporation Is a Potential Indicator of Tissue Strain and Subsequent Degeneration Following Experimental Traumatic Brain Injury. Clin. Biomech. (Bristol Avon).

[51] Cullen D.K., Vernekar V.N., LaPlaca M.C. (2011). Trauma-Induced Plasmalemma Disruptions in Three-Dimensional Neural Cultures Are Dependent on Strain Modality and Rate. J. Neurotrauma.

[52] Kilinc D., Gallo G., Barbee K. (2009). Mechanical Membrane Injury Induces Axonal Beading through Localized Activation of Calpain. Exp. Neurol..

[53] Park J., Jang S., Shin Y., Suh D., Park H. (2013). Calcium-Dependent Proteasome Activation Is Required for Axonal Neurofilament Degradation. Neural Regen. Res..

[54] Lucke-Wold B., Logsdon A., Nguyen L., Eltanahay A., Turner R., Bonasso P., Knotts C., Moeck A., Maroon J., Bailes J. (2018). Supplements, Nutrition, and Alternative Therapies for the Treatment of Traumatic Brain Injury. Nutr. Neurosci..

[55] Petronilho F., Feier G., de Souza B., Guglielmi C., Constantino L.S., Walz R., Quevedo J., Dal-Pizzol F. (2010). Oxidative Stress in Brain According to Traumatic Brain Injury Intensity. J. Surg. Res..

[56] Dong X., Wang Y., Qin Z. (2009). Molecular Mechanisms of Excitotoxicity and Their Relevance to Pathogenesis of Neurodegenerative Diseases. Acta Pharmacol. Sin..

[57] Lewén A., Fujimura M., Sugawara T., Matz P., Copin J.-C., Chan P.H. (2016). Oxidative Stress–Dependent Release of Mitochondrial Cytochrome c after Traumatic Brain Injury. J. Cereb. Blood Flow Metab..

[58] Schimmel S., Acosta S., Lozano D. (2017). Neuroinflammation in Traumatic Brain Injury: A Chronic Response to an Acute Injury. Brain Circ..

[59] Ryan K.C., Ashkavand Z., Norman K.R. (2020). The Role of Mitochondrial Calcium Homeostasis in Alzheimer’s and Related Diseases. Int. J. Mol. Sci..

[60] Denault J.-B., Salvesen G.S. (2001). Caspases. Curr. Protoc. Protein Sci..

[61] Liu W., Chen Y., Meng J., Wu M., Bi F., Chang C., Li H., Zhang L. (2018). Ablation of Caspase-1 Protects against TBI-Induced Pyroptosis in Vitro and in Vivo. J. Neuroinflamm..

[62] Abdul-Muneer P.M., Long M., Conte A.A., Santhakumar V., Pfister B.J. (2016). High Ca^2+^ Influx During Traumatic Brain Injury Leads to Caspase-1-Dependent Neuroinflammation and Cell Death. Mol. Neurobiol..

[63] McIlwain D.R., Berger T., Mak T.W. (2013). Caspase Functions in Cell Death and Disease. Cold Spring Harb. Perspect. Biol..

[64] Clark R., Kochanek P., Watkins S., Chen M., Dixon C., Seidberg N., Melick J., Loeffert J., Nathaniel P., Jin K. (2000). Caspase-3 Mediated Neuronal Death after Traumatic Brain Injury in Rats. J. Neurochem..

[65] Wang Y., Liu Y., Bi X., Baudry M. (2020). Calpain-1 and Calpain-2 in the Brain: New Evidence for a Critical Role of Calpain-2 in Neuronal Death. Cells.

[66] Saatman K.E., Creed J., Raghupathi R. (2010). Calpain as a Therapeutic Target in Traumatic Brain Injury. Neurotherapeutics.

[67] Wang Y., Briz V., Chishti A., Bi X., Baudry M. (2013). Distinct Roles for μ-Calpain and m-Calpain in Synaptic NMDAR-Mediated Neuroprotection and Extrasynaptic NMDAR-Mediated Neurodegeneration. J. Neurosci..

[68] Xu J., Kurup P., Zhang Y., Goebel-Goody S.M., Wu P.H., Hawasli A.H., Baum M.L., Bibb J.A., Lombroso P.J. (2009). Extrasynaptic NMDA Receptors Couple Preferentially to Excitotoxicity via Calpain-Mediated Cleavage of STEP. J. Neurosci..

[69] Wang Z.F., Gao C., Chen W., Gao Y., Wang H.C., Meng Y., Luo C.L., Zhang M.Y., Chen G., Chen X.P. (2019). Salubrinal Offers Neuroprotection through Suppressing Endoplasmic Reticulum Stress, Autophagy and Apoptosis in a Mouse Traumatic Brain Injury Model. Neurobiol. Learn. Mem..

[70] Liu S., Jin R., Xiao A., Chen R., Li J., Zhong W., Feng X., Li J. (2019). Induction of Neuronal PI3Kγ Contributes to Endoplasmic Reticulum Stress and Long-Term Functional Impairment in a Murine Model of Traumatic Brain Injury. Neurother. J. Am. Soc. Exp. NeuroTher..

[71] Sun D., Wang J., Liu X., Fan Y., Yang M., Zhang J. (2020). Dexmedetomidine Attenuates Endoplasmic Reticulum Stress-Induced Apoptosis and Improves Neuronal Function after Traumatic Brain Injury in Mice. Brain Res..

[72] Gage F. (2000). Mammalian Neural Stem Cells. Science.

[73] Hall E.D., Sullivan P.G., Gibson T.R., Pavel K.M., Thompson B.M., Scheff S.W. (2005). Spatial and Temporal Characteristics of Neurodegeneration after Controlled Cortical Impact in Mice: More than a Focal Brain Injury. J. Neurotrauma.

[74] Thompson H.J., Lifshitz J., Marklund N., Grady M.S., Graham D.I., Hovda D.A., McIntosh T.K. (2005). Lateral Fluid Percussion Brain Injury: A 15-Year Review and Evaluation. J. Neurotrauma.

[75] Mehrholz J., Major Y., Meissner D., Sandi-Gahun S., Koch R., Pohl M. (2005). The Influence of Contractures and Variation in Measurement Stretching Velocity on the Reliability of the Modified Ashworth Scale in Patients with Severe Brain Injury. Clin. Rehabil..

[76] Gao X., Deng-Bryant Y., Cho W., Carrico K., Hall E., Chen J. (2008). Selective Death of Newborn Neurons in Hippocampal Dentate Gyrus Following Moderate Experimental Traumatic Brain Injury. J. Neurosci. Res..

[77] Deng W., Aimone J.B., Gage F.H. (2010). New Neurons and New Memories: How Does Adult Hippocampal Neurogenesis Affect Learning and Memory?. Nat. Rev. Neurosci..

[78] Dupret D., Revest J.-M., Koehl M., Ichas F., de Giorgi F., Costet P., Abrous D.N., Piazza P.V. (2008). Spatial Relational Memory Requires Hippocampal Adult Neurogenesis. PLoS ONE.

[79] Zhou H., Chen L., Gao X., Luo B., Chen J. (2012). Moderate Traumatic Brain Injury Triggers Rapid Necrotic Death of Immature Neurons in the Hippocampus. J. Neuropathol. Exp. Neurol..

[80] Anderson K., Miller K., Fugaccia I., Scheff S. (2005). Regional Distribution of Fluoro-Jade B Staining in the Hippocampus Following Traumatic Brain Injury. Exp. Neurol..

[81] Sato M., Chang E., Igarashi T., Noble L. (2001). Neuronal Injury and Loss after Traumatic Brain Injury: Time Course and Regional Variability. Brain Res..

[82] Zhao Y., Xiang Q., Shi S., Li S., Tan L., Wang J., Jin X., Luo A. (2011). GABAergic Excitotoxicity Injury of the Immature Hippocampal Pyramidal Neurons’ Exposure to Isoflurane. Anesth. Analg..

[83] Hood K.N., Zhao J., Redell J.B., Hylin M.J., Harris B., Perez A., Moore A.N., Dash P.K. (2018). Endoplasmic Reticulum Stress Contributes to the Loss of Newborn Hippocampal Neurons after Traumatic Brain Injury. J. Neurosci..

[84] Redell J.B., Maynard M.E., Underwood E.L., Vita S.M., Dash P.K., Kobori N. (2020). Traumatic Brain Injury and Hippocampal Neurogenesis: Functional Implications. Exp. Neurol..

[85] Liu Y.-W., Li S., Dai S.-S. (2018). Neutrophils in Traumatic Brain Injury (TBI): Friend or Foe?. J. Neuroinflamm..

[86] Shi K., Zhang J., Dong J., Shi F.-D. (2019). Dissemination of Brain Inflammation in Traumatic Brain Injury. Cell. Mol. Immunol..

[87] Szmydynger-Chodobska J., Strazielle N., Gandy J.R., Keefe T.H., Zink B.J., Ghersi-Egea J.-F., Chodobski A. (2011). Posttraumatic Invasion of Monocytes across the Blood—Cerebrospinal Fluid Barrier. J. Cereb. Blood Flow Metab..

[88] Corps K.N., Roth T.L., McGavern D.B. (2015). Inflammation and Neuroprotection in Traumatic Brain Injury. JAMA Neurol..

[89] Hu Y., Mai W., Chen L., Cao K., Zhang B., Zhang Z., Liu Y., Lou H., Duan S., Gao Z. (2020). MTOR-Mediated Metabolic Reprogramming Shapes Distinct Microglia Functions in Response to Lipopolysaccharide and ATP. Glia.

[90] Carpentier P.A., Begolka W.S., Olson J.K., Elhofy A., Karpus W.J., Miller S.D. (2005). Differential Activation of Astrocytes by Innate and Adaptive Immune Stimuli. Glia.

[91] An Y., Chen Q., Quan N. (2011). Interleukin-1 Exerts Distinct Actions on Different Cell Types of the Brain in Vitro. J. Inflamm. Res..

[92] Ghafourifar P., Bringold U., Klein S.D., Richter C. (2001). Mitochondrial Nitric Oxide Synthase, Oxidative Stress and Apoptosis. Neurosignals.

[93] Izzy S., Liu Q., Fang Z., Lule S., Wu L., Chung J.Y., Sarro-Schwartz A., Brown-Whalen A., Perner C., Hickman S.E. (2019). Time-Dependent Changes in Microglia Transcriptional Networks Following Traumatic Brain Injury. Front. Cell. Neurosci..

[94] Witcher K.G., Bray C.E., Dziabis J.E., McKim D.B., Benner B.N., Rowe R.K., Kokiko-Cochran O.N., Popovich P.G., Lifshitz J., Eiferman D.S. (2018). Traumatic Brain Injury-Induced Neuronal Damage in the Somatosensory Cortex Causes Formation of Rod-Shaped Microglia That Promote Astrogliosis and Persistent Neuroinflammation. Glia.

[95] Dinet V., Petry K.G., Badaut J. (2019). Brain–Immune Interactions and Neuroinflammation After Traumatic Brain Injury. Front. Neurosci..

[96] Kumar A., Stoica B.A., Loane D.J., Yang M., Abulwerdi G., Khan N., Kumar A., Thom S.R., Faden A.I. (2017). Microglial-Derived Microparticles Mediate Neuroinflammation after Traumatic Brain Injury. J. Neuroinflamm..

[97] Fei M., Wang H., Zhou M., Deng C., Zhang L., Han Y. (2020). Podoplanin Influences the Inflammatory Phenotypes and Mobility of Microglia in Traumatic Brain Injury. Biochem. Biophys. Res. Commun..

[98] Gentleman S.M., Leclercq P.D., Moyes L., Graham D.I., Smith C., Griffin W.S.T., Nicoll J.A.R. (2004). Long-Term Intracerebral Inflammatory Response after Traumatic Brain Injury. Forensic Sci. Int..

[99] Ndode-Ekane X.E., Matthiesen L., Bañuelos-Cabrera I., Palminha C.A.P., Pitkänen A. (2018). T-Cell Infiltration into the Perilesional Cortex Is Long-Lasting and Associates with Poor Somatomotor Recovery after Experimental Traumatic Brain Injury. Restor. Neurol. Neurosci..

[100] Clausen F., Lorant T., Lewén A., Hillered L. (2007). T Lymphocyte Trafficking: A Novel Target for Neuroprotection in Traumatic Brain Injury. J. Neurotrauma.

[101] Newell-Rogers M.K., Rogers S.K., Tobin R.P., Mukherjee S., Shapiro L.A. (2020). Antagonism of Macrophage Migration Inhibitory Factory (MIF) after Traumatic Brain Injury Ameliorates Astrocytosis and Peripheral Lymphocyte Activation and Expansion. Int. J. Mol. Sci..

[102] Daglas M., Draxler D.F., Ho H., McCutcheon F., Galle A., Au A.E., Larsson P., Gregory J., Alderuccio F., Sashindranath M. (2019). Activated CD8 + T Cells Cause Long-Term Neurological Impairment after Traumatic Brain Injury in Mice. Cell Rep..

[103] Raad M., Nohra E., Chams N., Itani M., Talih F., Mondello S., Kobeissy F. (2014). Autoantibodies in Traumatic Brain Injury and Central Nervous System Trauma. Neuroscience.

[104] Sajja V.S.S.S., Hlavac N., VandeVord P.J. (2016). Role of Glia in Memory Deficits Following Traumatic Brain Injury: Biomarkers of Glia Dysfunction. Front. Integr. Neurosci..

[105] González-Reyes R.E., Nava-Mesa M.O., Vargas-Sánchez K., Ariza-Salamanca D., Mora-Muñoz L. (2017). Involvement of Astrocytes in Alzheimer’s Disease from a Neuroinflammatory and Oxidative Stress Perspective. Front. Mol. Neurosci..

[106] Jassam Y.N., Izzy S., Whalen M., McGavern D.B., el Khoury J. (2017). Neuroimmunology of Traumatic Brain Injury: Time for a Paradigm Shift. Neuron.

[107] Liddelow S.A., Guttenplan K.A., Clarke L.E., Bennett F.C., Bohlen C.J., Schirmer L., Bennett M.L., Münch A.E., Chung W.-S., Peterson T.C. (2017). Neurotoxic Reactive Astrocytes Are Induced by Activated Microglia. Nature.

[108] Dorsett C., McGuire J., Niedzielko T., DePasquale E., Meller J., Floyd C., McCullumsmith R. (2017). Traumatic Brain Injury Induces Alterations in Cortical Glutamate Uptake without a Reduction in Glutamate Transporter-1 Protein Expression. J. Neurotrauma.

[109] Pajarillo E., Rizor A., Lee J., Aschner M., Lee E. (2019). The Role of Astrocytic Glutamate Transporters GLT-1 and GLAST in Neurological Disorders: Potential Targets for Neurotherapeutics. Neuropharmacology.

[110] Gupta R., Prasad S. (2013). Early down Regulation of the Glial Kir4.1 and GLT-1 Expression in Pericontusional Cortex of the Old Male Mice Subjected to Traumatic Brain Injury. Biogerontology.

[111] Temple M., Hamm R. (1996). Chronic, Post-Injury Administration of D-Cycloserine, an NMDA Partial Agonist, Enhances Cognitive Performance Following Experimental Brain Injury. Brain Res..

[112] Adeleye A., Shohami E., Nachman D., Alexandrovich A., Trembovler V., Yaka R., Shoshan Y., Dhawan J., Biegon A. (2010). D-Cycloserine Improves Functional Outcome after Traumatic Brain Injury with Wide Therapeutic Window. Eur. J. Pharmacol..

[113] Dempsey R., Başkaya M., Doğan A. (2000). Attenuation of Brain Edema, Blood-Brain Barrier Breakdown, and Injury Volume by Ifenprodil, a Polyamine-Site N-Methyl-D-Aspartate Receptor Antagonist, after Experimental Traumatic Brain Injury in Rats. Neurosurgery.

[114] Yurkewicz L., Weaver J., Bullock M.R., Marshall L.F. (2005). The Effect of the Selective NMDA Receptor Antagonist Traxoprodil in the Treatment of Traumatic Brain Injury. J. Neurotrauma.

[115] Frandsen A., Schousboe A. (1991). Dantrolene Prevents Glutamate Cytotoxicity and Ca2+ Release from Intracellular Stores in Cultured Cerebral Cortical Neurons. J. Neurochem..

[116] Wu F., Xu K., Lei L., Zhang K., Xia L., Zhang M., Teng C., Tong H., He Y., Xue Y. (2019). Vitamin B12 Enhances Nerve Repair and Improves Functional Recovery After Traumatic Brain Injury by Inhibiting ER Stress-Induced Neuron Injury. Front. Pharmacol..

[117] Zhang L., Xiao H., Yu X., Deng Y. (2020). Minocycline Attenuates Neurological Impairment and Regulates Iron Metabolism in a Rat Model of Traumatic Brain Injury. Arch. Biochem. Biophys..

[118] Qu W., Liu N.-K., Wu X., Wang Y., Xia Y., Sun Y., Lai Y., Li R., Shekhar A., Xu X.-M. (2020). Disrupting NNOS–PSD95 Interaction Improves Neurological and Cognitive Recoveries after Traumatic Brain Injury. Cereb. Cortex.

[119] Tchantchou F., Zhang Y. (2013). Selective Inhibition of Alpha/Beta-Hydrolase Domain 6 Attenuates Neurodegeneration, Alleviates Blood Brain Barrier Breakdown, and Improves Functional Recovery in a Mouse Model of Traumatic Brain Injury. J. Neurotrauma.

[120] Wang J., Wang H., Zhong W., Li N., Cong Z. (2012). Expression and Cell Distribution of Metabotropic Glutamate Receptor 5 in the Rat Cortex Following Traumatic Brain Injury. Brain Res..

[121] Feng D., Wang W., Dong Y., Wu L., Huang J., Ma Y., Zhang Z., Wu S., Gao G., Qin H. (2014). Ceftriaxone Alleviates Early Brain Injury after Subarachnoid Hemorrhage by Increasing Excitatory Amino Acid Transporter 2 Expression via the PI3K/Akt/NF-ΚB Signaling Pathway. Neuroscience.

[122] Kang D.-W., Adams J.B., Coleman D.M., Pollard E.L., Maldonado J., McDonough-Means S., Caporaso J.G., Krajmalnik-Brown R. (2019). Long-Term Benefit of Microbiota Transfer Therapy on Autism Symptoms and Gut Microbiota. Sci. Rep..

[123] Fan H., Tang H.-B., Chen Z., Wang H.-Q., Zhang L., Jiang Y., Li T., Yang C.-F., Wang X.-Y., Li X. (2020). Inhibiting HMGB1-RAGE Axis Prevents pro-Inflammatory Macrophages/Microglia Polarization and Affords Neuroprotection after Spinal Cord Injury. J. Neuroinflamm..

[124] Khazdouz M., Mazidi M., Ehsaei M., Ferns G., Kengne A.P., Norouzy A.-R. (2017). Impact of Zinc Supplementation on the Clinical Outcomes of Patients with Severe Head Trauma: A Double-Blind Randomized Clinical Trial. J. Diet Suppl..

[125] Zhang Y., Chopp M., Zhang Z., Katakowski M., Xin H., Qu C., Ali M., Mahmood A., Xiong Y. (2017). Systemic Administration of Cell-Free Exosomes Generated by Human Bone Marrow Derived Mesenchymal Stem Cells Cultured under 2D and 3D Conditions Improves Functional Recovery in Rats after Traumatic Brain Injury. Neurochem. Int..

[126] Wright D., Kellermann A., Hertzberg V., Clark P., Frankel M., Goldstein F., Salomone J., Dent L., Harris O., Ander D. (2007). ProTECT: A Randomized Clinical Trial of Progesterone for Acute Traumatic Brain Injury. Ann. Emerg. Med..

[127] Wright D.W., Yeatts S.D., Silbergleit R., Palesch Y.Y., Hertzberg V.S., Frankel M., Goldstein F.C., Caveney A.F., Howlett-Smith H., Bengelink E.M. (2014). Very Early Administration of Progesterone for Acute Traumatic Brain Injury. N. Engl. J. Med..

[128] Wang G.-H., Jiang Z.-L., Li Y.-C., Li X., Shi H., Gao Y.-Q., Vosler P.S., Chen J. (2011). Free-Radical Scavenger Edaravone Treatment Confers Neuroprotection Against Traumatic Brain Injury in Rats. J. Neurotrauma.

[129] Sun G., Miao Z., Ye Y., Zhao P., Fan L., Bao Z., Tu Y., Li C., Chao H., Xu X. (2020). Curcumin Alleviates Neuroinflammation, Enhances Hippocampal Neurogenesis, and Improves Spatial Memory after Traumatic Brain Injury. Brain Res. Bull..

[130] Giacino J.T., Whyte J., Bagiella E., Kalmar K., Childs N., Khademi A., Eifert B., Long D., Katz D.I., Cho S. (2012). Placebo-Controlled Trial of Amantadine for Severe Traumatic Brain Injury. N. Engl. J. Med..

[131] Carlson A.P., Abbas M., Alunday R.L., Qeadan F., Shuttleworth C.W. (2018). Spreading Depolarization in Acute Brain Injury Inhibited by Ketamine: A Prospective, Randomized, Multiple Crossover Trial. J. Neurosurg..

[132] Williams S.E. (2009). Amantadine Treatment Following Traumatic Brain Injury in Children. Brain Inj..

[133] Spritzer S., Kinney C., Condie J., Wellik K., Hoffman-Snyder C., Wingerchuk D., Demaerschalk B. (2015). Amantadine for Patients with Severe Traumatic Brain Injury: A Critically Appraised Topic. Neurology.

[134] Papadia S., Hardingham G. (2007). The Dichotomy of NMDA Receptor Signaling. Neurosci. A Rev. J. Bringing Neurobiol. Neurol. Psychiatry.

[135] Hardingham G.E. (2009). Coupling of the NMDA Receptor to Neuroprotective and Neurodestructive Events. Biochem. Soc. Trans..

[136] Morris G., Bullock R., Marshall S., Marmarou A., Maas A., Marshall L. (1999). Failure of the Competitive N-Methyl-D-Aspartate Antagonist Selfotel (CGS 19755) in the Treatment of Severe Head Injury: Results of Two Phase III Clinical Trials. The Selfotel Investigators. J. Neurosurg..

[137] Yi N., Zhou L., Wang X., Song J., Han H., Zhang T., Wang Y., Shi Q., Xu H., Liang Q. (2019). MK-801 Attenuates Lesion Expansion Following Acute Brain Injury in Rats: A Meta-Analysis. Neural Regen. Res..

[138] Hammond F., Sauve W., Ledon F., Davis C., Formella A. (2018). Safety, Tolerability, and Effectiveness of Dextromethorphan/Quinidine for Pseudobulbar Affect Among Study Participants With Traumatic Brain Injury: Results From the PRISM-II Open Label Study. PM R J. Inj. Funct. Rehabil..

[139] Biegon A., Fry P., Paden C., Alexandrovich A., Tsenter J., Shohami E. (2004). Dynamic Changes in N-Methyl-D-Aspartate Receptors after Closed Head Injury in Mice: Implications for Treatment of Neurological and Cognitive Deficits. Proc. Natl. Acad. Sci. USA.

[140] Zhang Y., Fan J., Gu L., Yang H., Zhan S., Zhang H. (2021). Metabotropic Glutamate Receptor 5 Inhibits α-Synuclein-Induced Microglia Inflammation to Protect from Neurotoxicity in Parkinson’s Disease. J. Neuroinflamm..

[141] Barnes S., Sheffler D., Semenova S., Cosford N., Bespalov A. (2018). Metabotropic Glutamate Receptor 5 as a Target for the Treatment of Depression and Smoking: Robust Preclinical Data but Inconclusive Clinical Efficacy. Biol. Psychiatry.

[142] Byrnes K.R., Loane D.J., Stoica B.A., Zhang J., Faden A.I. (2012). Delayed MGluR5 Activation Limits Neuroinflammation and Neurodegeneration after Traumatic Brain Injury. J. Neuroinflamm..

[143] Wang J., Wang H., Cong Z., Zhang X., Zhou X., Zhang D. (2013). Activation of Metabotropic Glutamate Receptor 5 Reduces the Secondary Brain Injury after Traumatic Brain Injury in Rats. Biochem. Biophys. Res. Commun..

[144] Ngo V., Guerrero A., Lanum D., Burgett-Moreno M., Fenati G., Barr S., Neeki M. (2019). Emergent Treatment of Neuroleptic Malignant Syndrome Induced by Antipsychotic Monotherapy Using Dantrolene. Clin. Pract. Cases Emerg. Med..

[145] Muehlschlegel S., Sims J.R. (2008). Dantrolene: Mechanisms of Neuroprotection and Possible Clinical Applications in the Neurointensive Care Unit. Neurocrit. Care.

[146] Yano Y., Nakayama R., Imaizumi T., Terasaki H., Ushijima K. (2001). Dantrolene Ameliorates Delayed Cell Death and Concomitant DNA Fragmentation in the Rat Hippocampal CA1 Neurons Subjected to Mild Ischemia. Resuscitation.

[147] Wei H., Leeds P., Chen R., Wei W., Leng Y. (2000). Neuronal Apoptosis Induced by Pharmacological Concentrations of 3-Hydroxykynurenine: Characterization and Protection by Dantrolene and Bcl-2 Overexpression. J. Neurochem..

[148] Nakayama R., Yano T., Ushijima K., Abe E., Terasaki H. (2002). Effects of Dantrolene on Extracellular Glutamate Concentration and Neuronal Death in the Rat Hippocampal CA1 Region Subjected to Transient Ischemia. Anesthesiology.

[149] Mody I., MacDonald J. (1995). NMDA Receptor-Dependent Excitotoxicity: The Role of Intracellular Ca2+ Release. Trends Pharmacol. Sci..

[150] Tamaddonfard E., Farshid A., Samadi F., Eghdami K. (2014). Effect of Vitamin B12 on Functional Recovery and Histopathologic Changes of Tibial Nerve-Crushed Rats. Drug Res..

[151] Okada K., Tanaka H., Temporin K., Okamoto M., Kuroda Y. (2010). Methylcobalamin Increases Erk1/2 and Akt Activities through the Methylation Cycle and Promotes Nerve Regeneration in a Rat Sciatic Nerve Injury Model. Exp. Neurol..

[152] Suzuki K., Tanaka H., Ebara M., Uto K., Matsuoka H., Nishimoto S., Okada K., Murase T., Yoshikawa H. (2017). Electrospun Nanofiber Sheets Incorporating Methylcobalamin Promote Nerve Regeneration and Functional Recovery in a Rat Sciatic Nerve Crush Injury Model. Acta Biomater..

[153] Chen C.H., Huang Y.K., Jaw F.S. (2015). Ultrasound-Guided Perineural Vitamin B12 Injection for Peripheral Neuropathy. J. Med. Ultrasound.

[154] Sun H., Yang T., Li Q., Zhu Z., Wang L., Bai G., Li D., Li Q., Wang W. (2012). Experimetal Research Dexamethasone and Vitamin B12 Synergistically Promote Peripheral Nerve Regeneration in Rats by Upregulating the Expression of Brain-Derived Neurotrophic Factor. Arch. Med. Sci..

[155] Shakkour Z., Habashy K.J., Berro M., Takkoush S., Abdelhady S., Koleilat N., Eid A.H., Zibara K., Obeid M., Shear D. (2021). Drug Repurposing in Neurological Disorders: Implications for Neurotherapy in Traumatic Brain Injury. Neuroscientists.

[156] Lee S., Su Z., Emdad L., Gupta P., Sarkar D. (2008). Mechanism of Ceftriaxone Induction of Excitatory Amino Acid Transporter-2 Expression and Glutamate Uptake in Primary Human Astrocytes. J. Biol. Chem..

[157] Lim S., Su H., Nyam T., Chio C., Kuo J., Wang C. (2021). Ceftriaxone Therapy Attenuates Brain Trauma in Rats by Affecting Glutamate Transporters and Neuroinflammation and Not by Its Antibacterial Effects. BMC Neurosci..

[158] Tikka T., Fiebich B.L., Goldsteins G., Keinänen R., Koistinaho J. (2001). Minocycline, a Tetracycline Derivative, Is Neuroprotective against Excitotoxicity by Inhibiting Activation and Proliferation of Microglia. J. Neurosci..

[159] Sanchez Mejia R., Ona V., Li M., Friedlander R. (2001). Minocycline Reduces Traumatic Brain Injury-Mediated Caspase-1 Activation, Tissue Damage, and Neurological Dysfunction. Neurosurgery.

[160] Meythaler J., Fath J., Fuerst D., Zokary H. (2019). Safety and Feasibility of Minocycline in Treatment of Acute Traumatic Brain Injury. Brain Inj..

[161] Castillo J., Rama R., Dávalos A. (2000). Nitric Oxide–Related Brain Damage in Acute Ischemic Stroke. Stroke.

[162] Zhou L., Li F., Xu H.-B., Luo C.-X., Wu H.-Y., Zhu M.-M., Lu W., Ji X., Zhou Q.-G., Zhu D.-Y. (2010). Treatment of Cerebral Ischemia by Disrupting Ischemia-Induced Interaction of NNOS with PSD-95. Nat. Med..

[163] Sommer J.B., Bach A., Malá H., Strømgaard K., Mogensen J., Pickering D.S. (2017). In Vitro and in Vivo Effects of a Novel Dimeric Inhibitor of PSD-95 on Excitotoxicity and Functional Recovery after Experimental Traumatic Brain Injury. Eur. J. Neurosci..

[164] de Sousa M.C., Gjorgjieva M., Dolicka D., Sobolewski C., Foti M. (2019). Deciphering MiRNAs’ Action through MiRNA Editing. Int. J. Mol. Sci..

[165] Ge X.-T., Lei P., Wang H.-C., Zhang A.-L., Han Z.-L., Chen X., Li S.-H., Jiang R.-C., Kang C.-S., Zhang J.-N. (2014). MiR-21 Improves the Neurological Outcome after Traumatic Brain Injury in Rats. Sci. Rep..

[166] Wu J., He J., Tian X., Li H. (2020). Upregulation of MiRNA-9-5p Promotes Angiogenesis after Traumatic Brain Injury by Inhibiting Ptch-1. Neuroscience.

[167] Ghosh S., Garg S., Ghosh S. (2020). Cell-Derived Exosome Therapy: A Novel Approach to Treat Post-Traumatic Brain Injury Mediated Neural Injury. ACS Chem. Neurosci..

[168] Xin H., Li Y., Buller B., Katakowski M., Zhang Y., Wang X., Shang X., Zhang Z.G., Chopp M. (2012). Exosome-Mediated Transfer of MiR-133b from Multipotent Mesenchymal Stromal Cells to Neural Cells Contributes to Neurite Outgrowth. STEM CELLS.

[169] Wei J., Xiao G. (2013). The Neuroprotective Effects of Progesterone on Traumatic Brain Injury: Current Status and Future Prospects. Acta Pharmacol. Sin..

[170] Xiao G., Wei J., Yan W., Wang W., Lu Z. (2008). Improved Outcomes from the Administration of Progesterone for Patients with Acute Severe Traumatic Brain Injury: A Randomized Controlled Trial. Crit. Care (Lond. Engl.).

[171] Guo Q., Sayeed I., Baronne L., Hoffman S., Guennoun R., Stein D. (2006). Progesterone Administration Modulates AQP4 Expression and Edema after Traumatic Brain Injury in Male Rats. Exp. Neurol..

[172] Smith S. (1991). Progesterone Administration Attenuates Excitatory Amino Acid Responses of Cerebellar Purkinje Cells. Neuroscience.

[173] Cristino L., Bisogno T., di Marzo V. (2019). Cannabinoids and the Expanded Endocannabinoid System in Neurological Disorders. Nat. Rev. Neurol..

[174] Coomber B., O’Donoghue M.F., Mason R. (2008). Inhibition of Endocannabinoid Metabolism Attenuates Enhanced Hippocampal Neuronal Activity Induced by Kainic Acid. Synapse.

[175] Arizzi M., Cervone K., Aberman J., Betz A. (2004). Behavioral Effects of Inhibition of Cannabinoid Metabolism: The Amidase Inhibitor AM374 Enhances the Suppression of Lever Pressing Produced by Exogenously Administered Anandamide. Life Sci..

[176] Mukhopadhyay P., Pan H., Rajesh M., Bátkai S., Patel V., Harvey-White J., Mukhopadhyay B. (2010). CB1 Cannabinoid Receptors Promote Oxidative/Nitrosative Stress, Inflammation and Cell Death in a Murine Nephropathy Model. Br. J. Pharmacol..

[177] Heintz-Buschart A., Wilmes P. (2018). Human Gut Microbiome: Function Matters. Trends Microbiol..

[178] Lamichhane S., Sen P., Dickens A.M., Orešič M., Bertram H.C. (2018). Gut Metabolome Meets Microbiome: A Methodological Perspective to Understand the Relationship between Host and Microbe. Methods.

[179] Hayes C.L., Dong J., Galipeau H.J., Jury J., McCarville J., Huang X., Wang X.-Y., Naidoo A., Anbazhagan A.N., Libertucci J. (2018). Commensal Microbiota Induces Colonic Barrier Structure and Functions That Contribute to Homeostasis. Sci. Rep..

[180] Laurans L., Venteclef N., Haddad Y., Chajadine M., Alzaid F., Metghalchi S., Sovran B., Denis R.G.P., Dairou J., Cardellini M. (2018). Genetic Deficiency of Indoleamine 2,3-Dioxygenase Promotes Gut Microbiota-Mediated Metabolic Health. Nat. Med..

[181] Scheithauer T.P.M., Rampanelli E., Nieuwdorp M., Vallance B.A., Verchere C.B., van Raalte D.H., Herrema H. (2020). Gut Microbiota as a Trigger for Metabolic Inflammation in Obesity and Type 2 Diabetes. Front. Immunol..

[182] al Bander Z., Nitert M.D., Mousa A., Naderpoor N. (2020). The Gut Microbiota and Inflammation: An Overview. Int. J. Environ. Res. Public Health.

[183] Boulangé C.L., Neves A.L., Chilloux J., Nicholson J.K., Dumas M.E. (2016). Impact of the Gut Microbiota on Inflammation, Obesity, and Metabolic Disease. Genome Med..

[184] Brenner L.A., Stearns-Yoder K.A., Hoffberg A.S., Penzenik M.E., Starosta A.J., Hernández T.D., Hadidi D.A., Lowry C.A. (2017). Growing Literature but Limited Evidence: A Systematic Review Regarding Prebiotic and Probiotic Interventions for Those with Traumatic Brain Injury and/or Posttraumatic Stress Disorder. Brain Behav. Immun..

[185] Blander J.M., Longman R.S., Iliev I.D., Sonnenberg G.F., Artis D. (2017). Regulation of Inflammation by Microbiota Interactions with the Host. Nat. Immunol..

[186] Saglam E., Zırh S., Aktas C.C., Muftuoglu S.F., Bilginer B. (2021). Papaverine Provides Neuroprotection by Suppressing Neuroinflammation and Apoptosis in the Traumatic Brain Injury via RAGE- NF- B Pathway. J. Neuroimmunol..

[187] Hu Y., Sun Q., Li W., Zhang D., Ma B., Li S., Li W., Zhou M., Hang C. (2014). Biphasic Activation of Nuclear Factor Kappa B and Expression of P65 and C-Rel after Traumatic Brain Injury in Rats. Inflamm. Res. Off. J. Eur. Histamine Res. Soc..

[188] Yang K., Mu X.S., Hayes R.L. (1995). Increased Cortical Nuclear Factor-ΚB (NF-ΚB) DNA Binding Activity after Traumatic Brain Injury in Rats. Neurosci. Lett..

[189] Manivannan S., Marei O., Elalfy O., Zaben M. (2021). Neurogenesis after Traumatic Brain Injury—The Complex Role of HMGB1 and Neuroinflammation. Neuropharmacology.

[190] Andersson U., Yang H., Harris H. (2018). Extracellular HMGB1 as a Therapeutic Target in Inflammatory Diseases. Expert Opin. Ther. Targets.

[191] El-Far A.H., Sroga G., al Jaouni S.K., Mousa S.A. (2020). Role and Mechanisms of RAGE-Ligand Complexes and RAGE-Inhibitors in Cancer Progression. Int. J. Mol. Sci..

[192] Shen C.-Y., Lu C.-H., Wu C.-H., Li K.-J., Kuo Y.-M., Hsieh S.-C., Yu C.-L. (2020). The Development of Maillard Reaction, and Advanced Glycation End Product (AGE)-Receptor for AGE (RAGE) Signaling Inhibitors as Novel Therapeutic Strategies for Patients with AGE-Related Diseases. Molecules.

[193] Levenson C.W. (2020). Zinc and Traumatic Brain Injury: From Chelation to Supplementation. Med. Sci..

[194] Suh S., Chen J., Motamedi M., Bell B., Listiak K., Pons N., Danscher G., Frederickson C. (2000). Evidence That Synaptically-Released Zinc Contributes to Neuronal Injury after Traumatic Brain Injury. Brain Res..

[195] Hellmich H., Frederickson C., DeWitt D., Saban R., Parsley M., Stephenson R., Velasco M., Uchida T., Shimamura M., Prough D. (2004). Protective Effects of Zinc Chelation in Traumatic Brain Injury Correlate with Upregulation of Neuroprotective Genes in Rat Brain. Neurosci. Lett..

[196] Domínguez M., Blasco-Ibáñez J., Crespo C., Marquez Mari A., Martínez-Gui F. (2003). Zinc Chelation during Non-Lesioning Overexcitation Results in Neuronal Death in the Mouse Hippocampus. Neuroscience.

[197] Doering P., Stoltenberg M., Penkowa M., Rungby J., Larsen A., Danscher G. (2010). Chemical Blocking of Zinc Ions in CNS Increases Neuronal Damage Following Traumatic Brain Injury (TBI) in Mice. PLoS ONE.

[198] Levenson C.W. (2005). Zinc Supplementation: Neuroprotective or Neurotoxic?. Nutr. Rev..

[199] Suh S., Won S., Hamby A., Yoo B., Fan Y., Sheline C., Tamano H., Takeda A., Liu J. (2009). Decreased Brain Zinc Availability Reduces Hippocampal Neurogenesis in Mice and Rats. J. Cereb. Blood Flow Metab. Off. J. Int. Soc. Cereb. Blood Flow Metab..

[200] Adamo A.M., Oteiza P.I. (2010). Zinc Deficiency and Neurodevelopment: The Case of Neurons. BioFactors (Oxf. Engl.).

[201] Ismail H., Shakkour Z., Tabet M., Abdelhady S., Kobaisi A., Abedi R., Nasrallah L., Pintus G., Al-Dhaheri Y., Mondello S. (2020). Traumatic Brain Injury: Oxidative Stress and Novel Anti-Oxidants Such as Mitoquinone and Edaravone. Antioxidants.

[202] Zhang M., Teng C., Wu F., Ge L., Xiao J., Zhang H., Chen D. (2019). Edaravone Attenuates Traumatic Brain Injury through Anti-inflammatory and Anti-oxidative Modulation. Exp. Ther. Med..

[203] Hewlings S.J., Kalman D.S. (2017). Curcumin: A Review of Its Effects on Human Health. Foods.

[204] Xu D., Lian D., Wu J., Liu Y., Zhu M., Sun J., He D., Li L. (2017). Brain-Derived Neurotrophic Factor Reduces Inflammation and Hippocampal Apoptosis in Experimental Streptococcus Pneumoniae Meningitis. J. Neuroinflamm..

[205] Dai W., Wang H., Fang J., Zhu Y., Zhou J., Wang X., Zhou Y., Zhou M. (2018). Curcumin Provides Neuroprotection in Model of Traumatic Brain Injury via the Nrf2-ARE Signaling Pathway. Brain Res. Bull..

